# NSUN2 stimulates tumor progression via enhancing *TIAM2* mRNA stability in pancreatic cancer

**DOI:** 10.1038/s41420-023-01521-y

**Published:** 2023-07-01

**Authors:** Guizhen Zhang, Liwen Liu, Jianhao Li, Yu Chen, Yun Wang, Yize Zhang, Zihui Dong, Wenhua Xue, Ranran Sun, Guangying Cui

**Affiliations:** 1 Department of Infectious Diseases, The First Affiliated Hospital of Zhengzhou University, 450052 Zhengzhou, Henan P. R. China; 2 Gene Hospital of Henan Province, Precision Medicine Center, The First Affiliated Hospital of Zhengzhou University, 450052 Zhengzhou, Henan P. R. China; 3 Academy of Medical Sciences, Zhengzhou University, 450052 Zhengzhou, Henan P. R. China; 4 School of Basic Medical Sciences, Zhengzhou University, 450052 Zhengzhou, Henan P. R. China; 5 Department of Pharmacy, The First Affiliated Hospital of Zhengzhou University, 450052 Zhengzhou, Henan P.R. China

**Keywords:** Cancer, Epigenetics, Oncogenes

## Abstract

NSUN2 is a nuclear RNA methyltransferase which catalyzes 5-methylcytosine (m5C), a posttranscriptional RNA modification. Aberrant m5C modification has been implicated in the development of multiple malignancies. However, its function in pancreatic cancer (PC) needs to be elucidated. Herein, we determined that NSUN2 was overexpressed in PC tissues and related to aggressive clinical features. Silence of NSUN2 by lentivirus weakened the capability of proliferation, migration and invasion of PC cells in vitro and inhibited the growth and metastasis of xenograft tumors in vivo. Contrarily, overexpression of NSUN2 stimulated PC growth and metastasis. Mechanistically, m5C-sequencing (m5C-seq) and RNA-sequencing (RNA-seq) were carried out to identify downstream targets of NSUN2 and results showed that loss of NSUN2 led to decreased m5C modification level concomitant with reduced *TIAM2* mRNA expression. Further validation experiments proved that NSUN2 silence accelerated the decay of *TIAM2* mRNA in a YBX1-dependent manner. Additionally, NSUN2 exerted its oncogenic function partially through enhancing TIAM2 transcription. More importantly, disruption of the NSUN2/TIAM2 axis repressed the malignant phenotype of PC cells through blocking epithelial-mesenchymal transition (EMT). Collectively, our study highlighted the critical function of NSUN2 in PC and provided novel mechanistic insights into NSUN2/TIAM2 axis as promising therapeutic targets against PC.

## Introduction

Pancreatic cancer (PC), characterized by highly metastatic courses, is a fatal malignancy of the digestive system. The incidence of PC is stably increasing over the past decades, ranking the 12th most prevalent cancer worldwide [1, 2]. Despite improvements in diagnosis and treatment have been made, the prognosis of PC turns out to be disappointing, with an overall 5-year relative survival rate of 6% and the 7th leading cause of cancer-related mortality [2, 3]. The high incidence and mortality impose a great threat on human health and has become an enormous burden globally. Hence, it is imperative to figure out the molecular mechanisms underlying progression of PC and further identify promising targets for effective treatments.

Recently, RNA modification such as N6-methyladenine (m6A), 5-methylcytosine (m5C) and pseudouridine, has received considerable attention due to their fundamental role in cancer development [4, 5]. These aberrant epigenetic regulations in PC were also widely explored [6–9]. It has been elucidated that m6A modification of PIK3CB could promote PTEN-deficient PC progression by activating AKT signaling pathway [8]. Guo’s research revealed that m6A demethylase ALKBH5 serves as a tumor-suppressor in PC via activating PER1 in a posttranscriptional manner [9]. m5C, methylation of carbon 5 in cytosine, is another prevalent modification of RNA in eukaryotic cells. Similar with m6A, m5C modification is a dynamic reversible process, which can be catalyzed by the m5C methyltransferases (also called “writers”: NSUN, DNMT and TRDMT family members) and removed by the m5C demethylases (namely “erasers”: TET families) [10–12]. Moreover, m5C can be recognized by its binding proteins (“Readers”: ALYREF, YBX1) to affect RNA’s processing, including RNA export and stability [13, 14].

As the key m5C methyltransferase, NSUN2 (NOP2/Sun domain family, member 2) has been demonstrated to be overexpressed in a wide range of malignancies, including cancers of bladder, prostate, kidney, cervix, esophagus, stomach, liver, thyroid and breast [15]. Emerging data suggested that NSUN2 exerts a nonnegligible function in diverse biological process, such as cellular proliferation, differentiation, migration and involves in tumorigenesis in an m5C-dependent manner [16–19]. As elucidated by Su et al., NSUN2-mediated RNA m5C modification promoted esophageal squamous cell carcinoma (ESCC) progression through upregulating GRB2 by stabilizing its mRNA in an LIN28B-dependent manner [19]. Another study conducted by Lin et al. demonstrated that NSUN2 contributes to gastric cancer development by suppressing p57^Kip2^ [17]. However, the functional role of NSUN2 in PC is still obscure.

In this study, we identified that elevated expression of NSUN2 was correlated with unfavorable survival outcome in PC patients. Subsequent functional experiments revealed that the overexpression of NSUN2 could facilitate the proliferation, migration and invasion of PC. Mechanistically, TIAM2 was identified as the potential downstream target of NSUN2 using m5C-seq and RNA-seq. Further functional experiments confirmed that decreased TIAM2 could partially reverse the promotion effect of NSUN2 on the malignant phenotypes of PC. Additionally, NSUN2/TIAM2 axis enhanced epithelial-mesenchymal transition (EMT) process. Collectively, our study suggested that NSUN2 may be a promising prognostic marker and therapeutic target of PC.

## Results

### Differential expression profiles and prognostic value of m5C regulators in PC

**Fig. 1:**
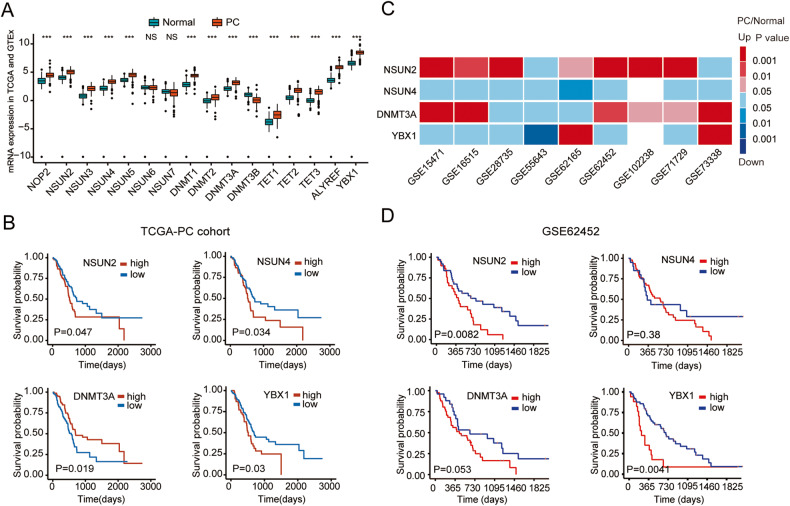
The altered expression profiles of m5C related genes in PC. **A** TCGA and GTEx databases were used to analyze the mRNA expression of m5C-related genes in PC tissues and normal pancreas tissues. ***P < 0.001. **B** Kaplan–Meier survival analysis was performed based TCGA database. **C** Heatmap showed the altered mRNA expression of m5C related genes in GEO datasets. Red indicates the related gene is upregulated and blue indicates the related gene is downregulated; White indicates that the related gene is absent in the dataset. **D** GSE62452 dataset was used for survival analysis in patients with low or high expression of NSUN2. NS, not significant.

To determine the expression profiles of m5C-related regulators in PC, the sequencing data of 179 PC samples and 171 control normal samples extracted from TCGA and GTEx was analyzed. Results demonstrated that m5C-related regulators were generally differentially expressed in PC tissues compared with normal tissues. Of these, 13 genes (*NOP2*, *NSUN2*, *NSUN3*, *NSUN4*, *NSUN5*, *DNMT1*, *DNMT2*, *DNMT3A*, *TET1*, *TET2*, *TET3*, *ALYREF* and *YBX1*) were aberrantly upregulated and *DNMT3B* was downregulated (Fig. 1A). Moreover, *NSUN2*, *NSUN4*, *DNMT3A* and *YBX1* were significantly correlated with the overall survival (OS) of PC patients (Fig. 1B, Fig. S1A). Subsequently, the aberrant expression of these four regulators was verified in 9 independent PC cohorts from the GEO database (Fig. 1C). However, only the overexpression of *NSUN2* and *YBX1* predicted a poor survival status of PC patients (Fig. 1D). Previous studies have demonstrated that overexpression of *YBX1* could accelerate PC growth and recognize m5C modification catalyzed by NSUN2, thereby promoting pathogenesis of bladder cancer [20, 21]. However, the role of NSUN2 in PC remains little known. Hence, we selected NSUN2 as our target gene for the subsequent experiment and determined whether NSUN2 exerts an oncogenic role in PC progression.

### NSUN2 is overexpressed in PC and correlated with an unfavorable prognosis

**Fig. 2:**
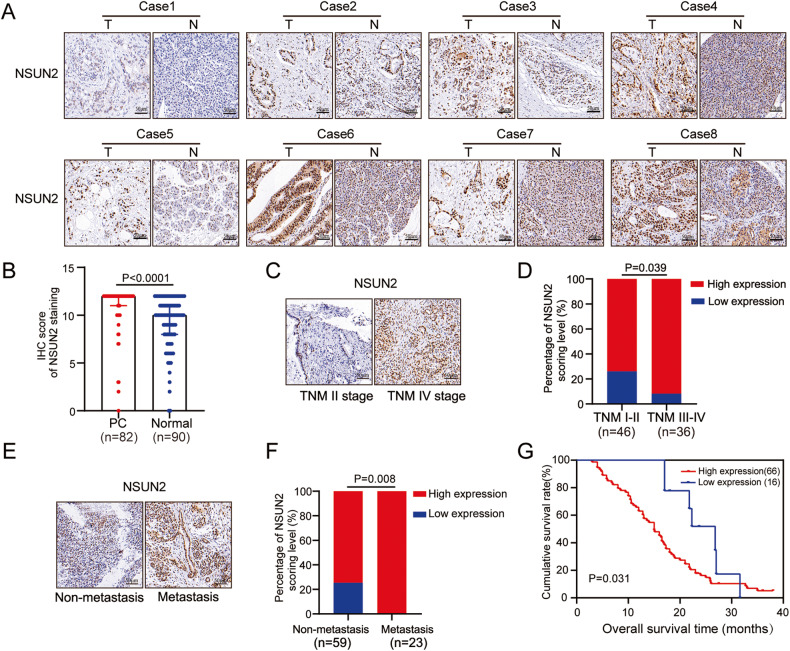
NSUN2 overexpression was determined by a PC tissue microarray and predicted poor prognosis. **A** Representative IHC staining images and **B** IHC scores of NSUN2 expression in paired PC tissues and adjacent non-tumor tissues. Scale bar = 50 μm. **C**–**F** NSUN2 overexpression in PC was associated with **C**, **D** advanced TNM stage and **E**, **F** metastasis. **G** Upregulation of NSUN2 was significantly associated with shorter OS in PC patients.

Next, the protein expression of *NSUN2* was further validated by IHC staining on a microarray containing 90 paired PC tissues and adjacent normal tissues. In accordance with the aforementioned data, aberrant overexpression of NSUN2 was observed in PC tissues (Fig. 2A, B). Moreover, correlations between NSUN2 expression and clinical characteristics of patients with PC were explored (Table 1). We observed that elevated NSUN2 expression indicated an advanced TNM stage (Fig. 2C, D) and distant metastasis (Fig. 2E, F) in PC. Consistently, Kaplan–Meier analysis revealed that patients with NSUN2 overexpression were prone to have a shorter OS time than those with low expression (median: 15.60 months vs. 21.78 months, Fig. 2G). However, NSUN2 expression was not identified as an independent risk factor for OS in PC by the multivariate Cox regression analysis in this cohort (Supplementary Table 1, Fig. S1B). Altogether, these results provide further strong evidence that NSUN2 upregulation is an unfavorable prognosticator for PC.

**Table 1:** Correlation of NSUN2 expression and clinical features in patients with PC.

Variables	NSUN2 expression		
	Low (*n* = 15)	High (*n* = 67)	*P* value
*Gender*			0.165
Male	11	36	
Female	4	31	
*Age(years)*			0.336
<60	6	36	
≥60	9	31	
*Tumor differentiation*			0.48
Poor	5	29	
Well	10	38	
*Tumor size (cm)*			0.581
<4	8	30	
≥4	7	36	
*Tumor number*			0.426
Single	10	36	
Multiple	1	12	
*TNM stage*			0.039*
I–II	12	34	
II–IV	3	33	
*Distant metastasis*			0.008*
M0	15	44	
M1	0	23	
*Nervous invasion*			0.357
Negative	4	26	
Positive	11	40	
*Venous invasion*			0.771
Negative	10	39	
Positive	5	27	
CA199 (U/ml)			0.441
<40	2	15	
≥40	11	51	
CA125 (U/ml)			0.709
<35	8	36	
≥35	2	17	
Survival time(months)	21.78(15.85–27.71)	15.60(7.00–24.20)	0.01*

Partial data were not available, and statistical analyses were based on available data.

*p < 0.05.

### NSUN2 accelerates the proliferation of PC cells both in vitro and in vivo

**Fig. 3:**
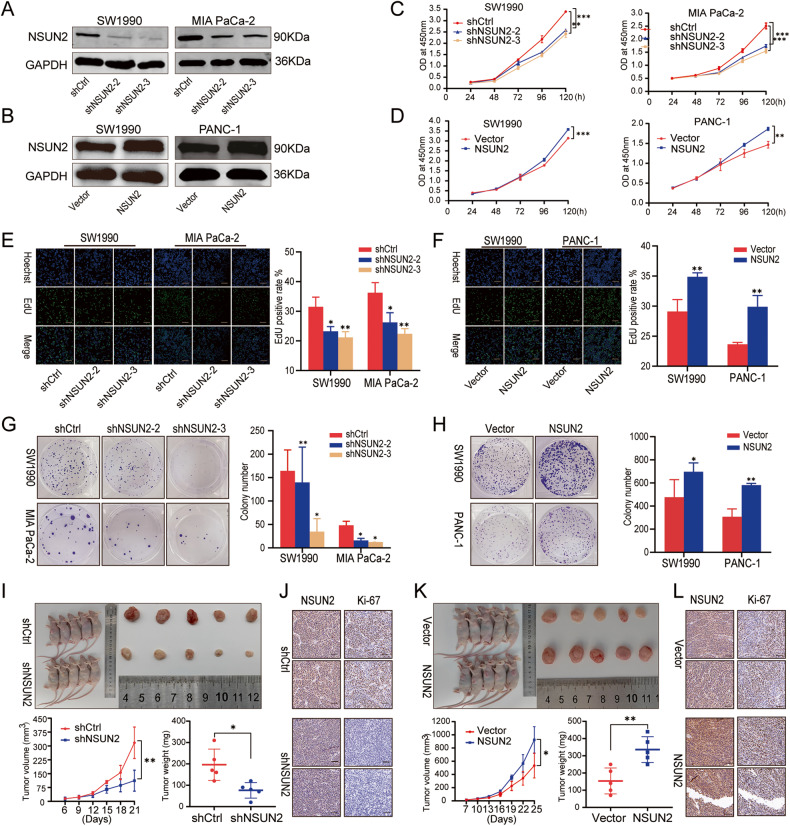
NSUN2 promotes the PC cell proliferation in vitro and accelerates tumor growth in vivo. **A**, **B** Western blot confirmed the knockdown and overexpression of NSUN2 by lentiviral constructs in PC cell lines. **C**, **D** Cell viability was determined by CCK-8 in cells with stable NSUN2 knockdown or overexpression. **E**, **F** EdU staining assays were performed to evaluate cell proliferation. **G**, **H** Colony formation assays. **I**, **K** Tumor growth and weight were monitored in mice. **J**, **L** IHC staining of NSUN2 and Ki-67 expression in tumor sections. Scale bar = 100 μm. Data are presented as the mean ± SD of at least 3 independent experiments; *p < 0.05, **p < 0.01, ***p < 0.001.

Then we explored the function of NSUN2 in PC cells. Firstly, NSUN2 protein expression was validated in the human pancreatic ductal epithelial cell line (HPDE6C7) and several PC cell lines (SW1990, PANC-1, CFPAC-I and MIA PaCa-2). With the exception of CFPAC-I cells, other PC cell lines all exhibited higher NSUN2 expression than HPDE6C7 (Fig. S2). Subsequently, we established cell lines with NSUN2 silence or overexpression by lentiviral transduction, and Western blot analysis proved the successful transduction (Fig. 3A, B). Functional experiments revealed that knockdown of NSUN2 substantially retarded PC cells proliferation as evidenced by CCK-8, EdU and colony formation assays (Fig. 3C, E, G), while opposite effects were observed in cells with NSUN2 upregulation (Fig. 3D, F, H).

To further determine the impact of NSUN2 on tumor growth in vivo, we established a subcutaneous xenograft model. It was verified that NSUN2-silenced cells formed smaller tumor nodules than those control cells formed (Fig. 3I), while upregulation of NSUN2 in PC cells significantly stimulated tumor growth as evidenced by an accelerated growth curve and increased tumor weight (Fig. 3K), which was consistent with our previous observation in vitro. Additionally, IHC staining of sections from the xenograft tumors showed a decreased portion of Ki-67-positive cells in NSUN2 silence group (Fig. 3J), but an increased portion in NSUN2 overexpression group (Fig. 3L). Taken together, these findings indicate that NSUN2 could promote the growth capacity of PC.

### NSUN2 modulates PC migration and invasion in vitro and metastasis in vivo

**Fig. 4:**
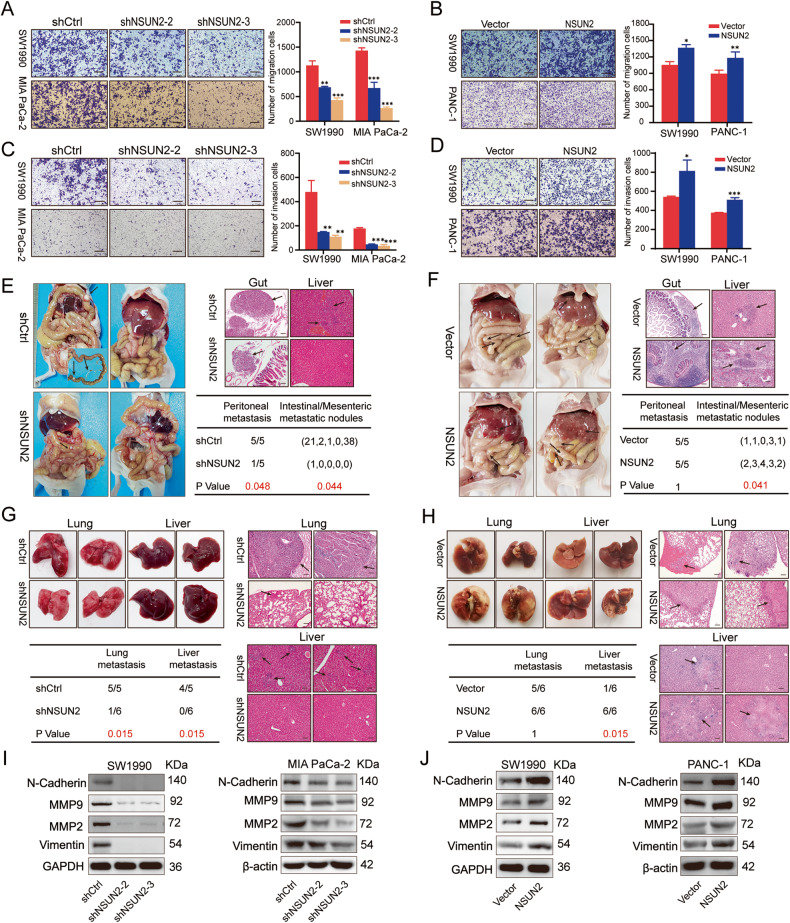
NSUN2 induces migration and invasion of PC cells in vitro and facilitates metastasis in vivo. **A**, **B** Migration ability and **C**, **D** invasion ability of PC cells with NSUN2 silence or overexpression were evaluated by Transwell assays. Data are shown as the mean ± SD of 3 replicates. *P < 0.05, **P < 0.01, ***P < 0.001. **E**, **F** Representative images of tumor peritoneal dissemination and statistical data were indicated. **G**, **H** Representative images of hematogenous metastasis model established by injecting PC cells through the tail vein. Scale bar = 100 μm. **I**, **J** Altered expression of EMT-associated genes in cells with NSUN2 silence or overexpression. Scale bar = 100 μm.

Subsequently, we further explored whether NSUN2 regulates the motility of PC cells via Transwell migration and invasion assays. Results displayed that attenuation of NSUN2 expression markedly impaired the migratory and invasive ability of SW1990 and MIA PaCa-2 cells (Fig. 4A, C), whereas overexpression of NSUN2 was stimulative (Fig. 4B, D). Next, a peritoneal dissemination model and a hematogenous metastasis model were established to validate the role of NSUN2 on metastasis in vivo. Remarkably, extensive intestinal and mesenteric metastases, liver metastases, and ascites formation were observed in the control group, whereas only one mouse in the shNSUN2 group suffered intestinal metastasis (Fig. 4E). Although peritoneal dissemination occurred in all mice, the number of intestinal metastatic nodules formed in the NSUN2 overexpression group were statistically higher than the control vector group (Fig. 4F). Similarly, SW1990/shCtrl cells formed remarkably large and excessive lung and liver metastatic foci than NSUN2-silenced SW1990 cells (Fig. 4G), and more extensive liver metastases were observed in the NSUN2 overexpression group (Fig. 4H). Taken together, these findings suggest that NSUN2 could facilitate migration and invasion of PC cells in vitro and metastasis in vivo.

Considering the crucial role of EMT in cancer metastasis, we further investigated whether NSUN2 expression affected EMT. Interestingly, NSUN2-silenced SW1990 and MIA PaCa-2 cells exhibited a lower expression of N-cadherin, MMP9, MMP2 and vimentin than control cells (Fig. 4I). Conversely, enforced NSUN2 expression had the opposite effects (Fig. 4J). We also observed that silencing NSUN2 resulted in the formation of cobblestone-like PC cells (Fig. S3A), whereas cells with overexpressed NSUN2 exhibited a spindle-like shape (Fig. S3B). Based on these findings, it is plausible that NSUN2 may play a metastasis-prompting role in PC by inducing EMT.

### Characterization of mRNA m5C profile by m5C-seq in PC cells

**Fig. 5:**
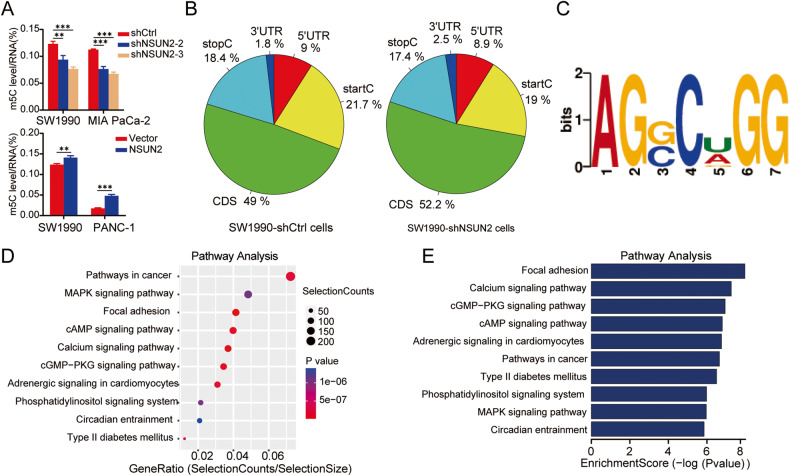
Characterization of mRNA m5C in PC cells. **A** Relative m5C levels were assessed in cells with or without NSUN2 silence or overexpression, respectively. **B** The distribution of m5C sites within distinct mRNA regions. **C** The preferred sequence context of m5C sites in PC cells. **D**, **E** KEGG analysis for genes with downregulated m5C modification after knockdown of NSUN2. The ten most enriched pathways are shown (for full list see Supplementary Table 6).

Given that NSUN2 is one of the m5C RNA methyltransferases, we explored whether NSUN2 affected the global RNA m5C modification level of PC cells. As anticipated, NSUN2-silenced groups showed decreased methylation levels, and increased levels were observed in NSUN2-overexpressing cells (Fig. 5A), indicating the m5C methyltransferase activity of NSUN2 in PC cells. Next, m5C-seq assays were performed to investigate the effect of NSUN2 silencing on the m5C profile of PC cells. Results showed that m5C sites were distributed in all regions of the mRNA, and the distribution patterns in two groups were similar (Fig. 5B). Of note, in addition to about half of m5C sites distributed within CDS, the region near the start codon is also an important source of m5C peaks, suggesting that m5C may be involved in translation regulation. These observations accord with previously published reports [14, 22]. Moreover, we also investigated the sequence context of methylated regions using DREME software. “AGSCDGG” (S “Strong” = C/G, D “Not C” = A/G/U) was identified as the preferred motif in PC cells with the lowest E-value (4.1e−197) (Fig. 5C). Although this sequence context was not exactly consistent with the preferred motif previously measured by bisulfite sequencing, it also demonstrated that m5C sites are embedded in CG-rich environments [22, 23].

To get insights into the regulatory role of m5C, we performed KEGG analysis on mRNAs with differentially downregulated m5C sites after NSUN2 silencing and observed that they were mainly enriched in pathways in cancer, MAPK pathways, focal adhesion and other oncogenic pathways (Fig. 5D, E), which suggests that NSUN2 may be involved in cell proliferation and migration in an m5C-dependent manner.

### TIAM2 was identified as a target of NSUN2 by m5C-seq and RNA-seq in PC

**Fig. 6:**
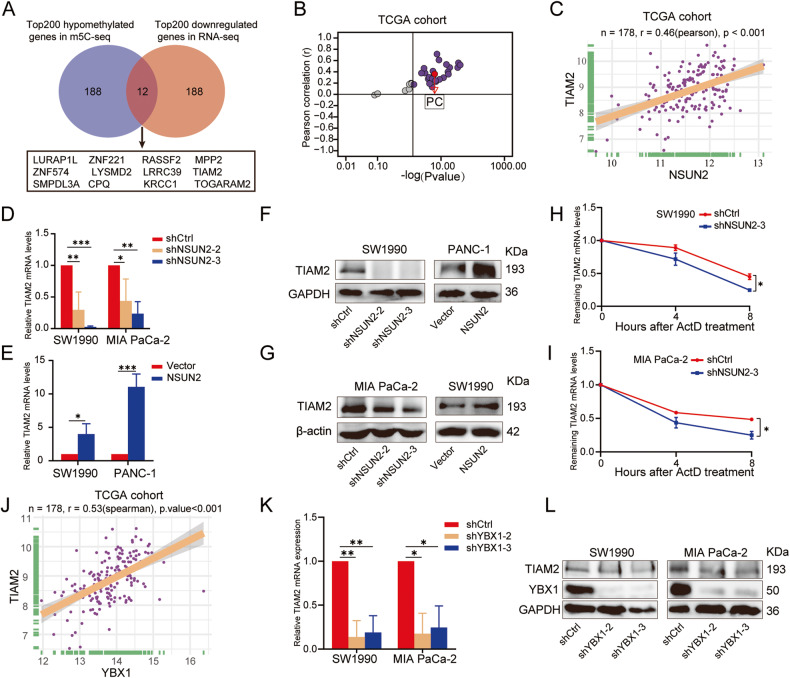
TIAM2 was identified as a downstream target of NSUN2. **A** Venn diagram showed the selection for the downstream target of NSUN2 according to RNA-seq and m5C-seq. **B**, **C** *TIAM2* expression was positively correlated with *NSUN2* expression in PC based on TCGA data. **D**–**G** *TIAM2* expression upon NSUN2 knockdown or overexpression were determined by **D**, **E** qRT-PCR and **F**, **G** Western blot. **H**, **I** The relative *TIAM2* mRNA expression upon NSUN2 knockdown in SW1990 and MIA PaCa-2 cells treated with 5 μg/ml actinomycin D for indicated times. Data are presented as the mean ± SD of 2 replicates. **J** A positive correlation between *TIAM2* and *YBX1* mRNA expression in PC based on TCGA data. **K**, **L** *TIAM2* mRNA(**K**) and protein expression(**L**) in SW1990 and MIA PaCa-2 cells transfected with YBX1 shRNA and the corresponding control lentivirus. Data are presented as the mean ± SD of 3 independent experiments. *P < 0.05, **P < 0.01, ***P < 0.001.

It has been confirmed that enhanced mRNA transcription is positively correlated with NSUN2 mediated-m5C modification and consequently achieved by m5C reader YBX1 through maintaining mRNA stability [21]. Considering the aberrant expression and prognostic role of NSUN2 and YBX1 in PC patients (Fig. 1A–D), we hypothesized that similar regulatory mechanisms may exist in PC. Therefore, we analyzed m5C-seq and RNA-seq data in PC cells to excavate the potential targets of NSUN2 in PC. Results demonstrated that among 2099 annotated genes with 4138 differential hypomethylated m5C sites, 1387 (66.08%) genes were downregulated, and 712 (33.92%) genes were upregulated in mRNA level (Supplementary Table 2). Twelve potential targets (*LURAP1L*, *ZNF221*, *RASSF2*, *MPP2*, *ZNF574*, *LYSMD2*, *LRRC39*, *TIAM2*, *SMPDL3A*, *CPQ*, *KRCC1*, *TOGARAM2*), which were downregulated in mRNA level with top hypomethylated sites, were screened out (Fig. 6A and Table 2). Of these, *TIAM2*, *LURAP1L*, *ZNF574* and *KRCC1* were upregulated in PC and positively correlated with NSUN2 (Fig. 6B, C, Fig. S4A, B), but only *TIAM2* had a statistically significant impact on both OS and relapse-free survival (RFS) in PC patients (Fig. S5A, B).

**Table 2:** Hypomethylated m5C sites in 12 candidate genes.

chrom	txStart	txEnd	PeakID	Peak_length	transcript_id	GeneName	Foldchange	*P*_value
chr1	100160465	100160571	diffreps_peak_137168	106	ENST00000342895	LRRC39	775.60	0
chr2	88055641	88055729	diffreps_peak_1657499	88	ENST00000347055	KRCC1	439.90	6.21725E−15
chr2	29060301	29060860	diffreps_peak_1594275	559	ENST00000379558	TOGARAM2	581.90	0
chr2	28998142	28998253	diffreps_peak_1594155	111	ENST00000379558	TOGARAM2	9.26	1.41337E−09
chr2	28970938	28971114	diffreps_peak_1594108	176	ENST00000420297	TOGARAM2	259.20	2.19326E−09
chr2	29035463	29035656	diffreps_peak_1594234	193	ENST00000379558	TOGARAM2	5.92	1.4855E−09
chr6	155129981	155130417	diffreps_peak_2704481	436	ENST00000360366	TIAM2	724.00	0
chr6	155183236	155183500	diffreps_peak_2704545	264	ENST00000360366	TIAM2	556.10	0
chr6	155216636	155217090	diffreps_peak_2704569	454	ENST00000275246	TIAM2	20.13	0
chr6	122789048	122789220	diffreps_peak_2679261	172	ENST00000539041	SMPDL3A	362.50	1.4525E−12
chr8	97029402	97029494	diffreps_peak_3047253	92	ENST00000220763	CPQ	439.90	6.21725E−15
chr9	12821385	12821800	diffreps_peak_3115140	415	ENST00000319264	LURAP1L	594.90	0
chr15	51737621	51738137	diffreps_peak_1013885	516	ENST00000267838	LYSMD2	827.20	0
chr17	43900470	43900598	diffreps_peak_1294679	128	ENST00000377184	MPP2	297.90	1.40044E−10
chr17	43907521	43907942	diffreps_peak_1294695	421	ENST00000612133	MPP2	414.10	3.79696E−14
chr19	42074904	42075246	diffreps_peak_1520673	342	ENST00000597391	ZNF574	3.65	0
chr19	42068476	42068580	diffreps_peak_1520658	104	ENST00000222339	ZNF574	517.40	0
chr19	42070365	42071000	diffreps_peak_1520665	635	ENST00000600245	ZNF574	259.20	2.19326E−09
chr19	43960186	43960340	diffreps_peak_1524267	154	ENST00000251269	ZNF221	388.30	2.34701E−13
chr20	4815101	4815123	diffreps_peak_1809935	22	ENST00000379376	RASSF2	465.80	9.99201E−16
chr20	4798009	4798085	diffreps_peak_1809908	76	ENST00000379376	RASSF2	207.60	8.73805E−08

TIAM2, namely T-cell lymphoma invasion and metastasis 2, is a Rac1-Guanine nucleotide exchange factor (Rac1-GEF), which facilitates the exchange of GDP for GTP, thereby activating Rac1 [24]. It has been demonstrated that TIAM2 could facilitate cancer cells proliferation and migration, thereby facilitating cancer progression [25–27]. Although very limited evidence suggested that TIAM2 is correlated with poor prognosis in PC [28], its regulatory mechanism remains unclear. Therefore, we selected *TIAM2* as a candidate target of NSUN2 for further investigation. Strikingly altered TIAM2 mRNA (Fig. 6D, E) and protein (Fig. 6F, G) expression upon NSUN2 silencing or overexpression further supported our postulation.

It has been validated that NSUN2 could affect metabolism of mRNA such as degradation and nuclear export [14, 18, 21]. We further investigated whether NSUN2 regulates TIAM2 expression by affecting its mRNA stability. Actinomycin D assay showed that the stability of *TIAM2* mRNA was significantly reduced upon NSUN2 silence (Fig. 6H, I). Although there was no significant difference in the stability of *TIAM2* mRNA compared to the control group, possibly due to the high level of endogenous NSUN2 expression in SW1990 cells, we still observed enhanced stability of *TIAM2* mRNA in PANC-1 cells with NSUN2 overexpression (Fig. S6A, B).

The stability of m5C-modified mRNAs was preferentially positively regulated by the YBX1(m5C reader) [13, 21]. Therefore, we further explored whether YBX1 influences the expression of TIAM2 in PC. Based on the TCGA database, a positive correlation between *YBX1* and *TIAM2* mRNA expression in PC was identified (Fig. 6J). In line with this data, we observed that the transduction of shRNAs targeting YBX1 led to the reduction of *TIAM2* mRNA stability (Fig. S6C, D) and reduced the expression of TIAM2 (Fig. 6K, L). Moreover, YBX1 plasmids were transfected into the control cells and NSUN2 silenced cells, respectively. We observed that overexpression of YBX1 significantly upregulated the expression of TIAM2 in the control cells. However, forced expression of YBX1 in NSUN2 silenced cells only slightly restored the downregulation of TIAM2 induced by NSUN2 knockdown (Fig. S6E, F, G), suggesting that YBX1 may regulate TIAM2 expression through NSUN2-mediated m5C modification.

### TIAM2 is responsible for NSUN2-mediated PC progression

**Fig. 7:**
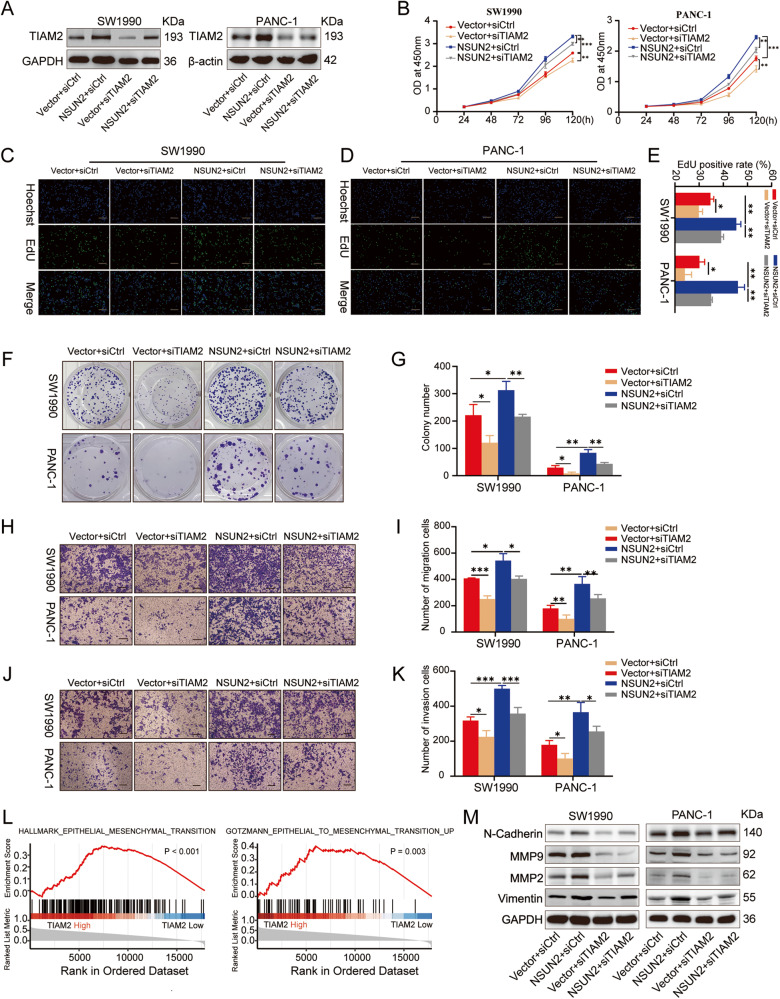
TIAM2 mediates NSUN2-induced cell proliferation, migration and invasion of PC cells. **A** Western blot was conducted to examine TIAM2 expression in the Vector+siCtrl, NSUN2+siCtrl, Vector+siTIAM2, NSUN2+siTIAM2 cells. **B** Cell viability of indicated cells was determined by CCK-8 assays. **C**–**E** EdU staining assays for indicated cells. **F**, **G** The ability of colonies formation for indicated cells. **H-I** The migration ability and **J**, **K** invasion ability of indicated cells was evaluated by Transwell assays. **L** GSEA indicated that EMT pathway was enriched in the patients with high TIAM2 expression. **M** TIAM2 silence could suppress EMT-related genes expression and rescue these genes expression upregulated by NSUN2. Scale bar = 100 μm. Data are shown as the mean ± SD of 3 independent experiments. *P < 0.05, **P < 0.01, ***P < 0.001.

To further investigate the impact of TIAM2 on NSUN2-induced cancer progression, we co-transfected siRNA (siTIAM2 or siCtrl) into vector control or NSUN2-overexpressing PC cells. The transfection efficiency was confirmed by Western blot (Fig. 7A). Intriguingly, compared with the vector+siCtrl group, cells in vector+siTIAM2 group exhibited lower proliferation capacity and formed less and smaller colonies (Fig. 7B–G). Additionally, impaired migratory and invasive abilities were observed in TIAM2-silencing cells (Fig. 7H–K). Most notably, TIAM2 downregulation largely abolished the promoting effects of NSUN2 overexpression on abovementioned malignant phenotypes (Fig. 7B–K). Subsequently, we performed KEGG analysis and GSEA based on TCGA dataset. Results indicated that proliferation- and metastasis-related pathways such as EMT were significantly enriched in patients with high TIAM2 expression (Fig. 7L, Fig. S7). Further study displayed that TIAM2 silencing substantially decreased the expression of EMT-related molecules (N-cadherin, MMP9, MMP2, Vimentin) in PC cells, and partly reversed their expression in the presence of overexpressed-NSUN2 (Fig. 7M). These data provide compelling evidence that upregulation of TIAM2 by NSUN2-mediated m5C modification is significant for promoting PC progression.

### Clinical relevance of the NSUN2/TIAM2 axis in PC patients

**Fig. 8:**
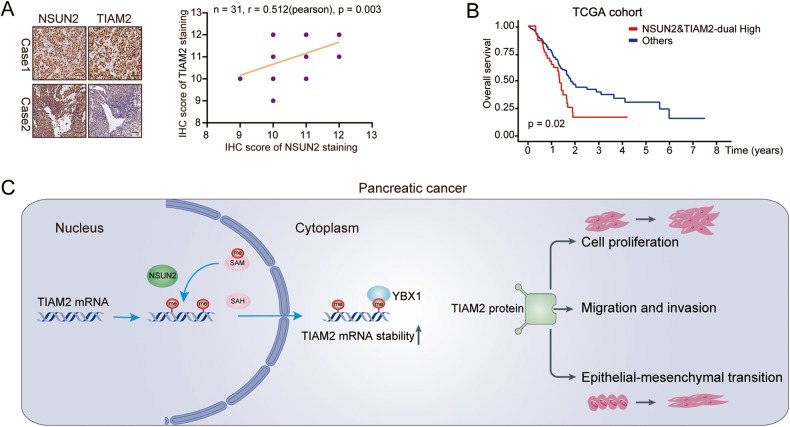
The clinical relevance and mechanism of NSUN2/TIAM2 axis in PC. **A** IHC staining of NSUN2 and TIAM2 in PC tissues and the positive correlation between them. (*n* = 31). Scale bar =100 μm. **B** Patients with high NSUN2 and high TIAM2 expression had poor overall survival. **C** The schematic figure shows that NSUN2 upregulates m5C level of *TIAM2* mRNA, and may sustain the stability of *TIAM2* mRNA; TIAM2 overexpression could promote cell proliferation, and activate EMT process in PC.

Eventually, we performed IHC assays to determine the expression of TIAM2 on PC tissues. IHC staining confirmed that TIAM2 can be mainly detected in the cell nuclei and also in the cytoplasm of PC cells. Additionally, a positive correlation between TIAM2 and NSUN2 was observed in PC tissues (Fig. 8A). Moreover, survival analysis based on TCGA dataset demonstrated that patients with high *NSUN2* and high *TIAM2* mRNA expression showed decreased overall survival than others (Fig. 8B, Fig. S8). Taken together, these results further underscore the notion that NSUN2 promotes PC tumorigenesis partially by enhancing TIAM2 expression and consequently activating EMT process. The suggested regulatory mechanism is illustrated in Fig. 8C.

## Discussion

Pancreatic cancer is a leading cause of cancer death worldwide. Despite advances in treatment, the prognosis of PC remains deadly poor. Thus, it’s urgently needed to investigate the underlying mechanisms related to PC progression and search for novel therapeutic strategies. m5C methylation is an abundant modification on eukaryotic RNA and emerging studies have demonstrated the m5C dysregulation are implicated in human diseases including cancers [29–32]. However, thus far, studies on m5C modification and its functions in PC are limited.

In this study, we determined that NSUN2 was overexpressed in PC tissues compared with normal tissues and its elevated expression indicated an unfavorable prognosis, which accords with the previous work [15, 23]. Functional experiments showed that NSUN2 could accelerate PC proliferation and promote migration and invasion in vitro, thereby enhancing tumor growth and metastasis in vivo. Consistent with our findings, it has been reported that NSUN2 is involved in the development of gastric cancer, esophageal squamous cell carcinoma (ESCC), gallbladder carcinoma and other human cancers [16, 17, 19, 33–35]. Lin et al.’s research showed that NSUN2 could be modulated by SUMO-2/3 and promote gastric cancer cell proliferation and motility [17]. Besides, NSUN2 has been proven to enhance the initiation and progression of ESCC by upregulating GRB2 expression [19]. Our findings further support the oncogenic role of NSUN2 in malignancies.

To further investigate the regulatory role of NSUN2 during PC progression, we conducted m5C quantification assays and found that NSUN2 silence in PC cells resulted in the significantly decreased m5C level. m5C-seq revealed the distribution patterns of m5C along mRNAs in PC. Complementary to the prior studies in which bisulfite sequencing was utilized, the most common motif “AGSCDGG” was identified by m5C-antibody based m5C-seq. KEGG analysis showed that differentially methylated sites-harboring genes were mainly enriched in several oncogenic pathways, which are closely related with tumor growth and metastasis [36, 37]. Subsequently, we identified *TIAM2* as a downstream target of NSUN2 by sequencing and bioinformatics analysis. Accumulating data showed that TIAM2 was involved in some tumor-associated biological processes, including immune response, cell proliferation, apoptosis, motility [38–43]. Recent studies showed that TIAM2 was frequently upregulated in various human malignancies such as liver cancer, lung cancer [25, 27, 44]. Importantly, Jiang and coworkers found that TIAM2 could exert a tumor-promoting effect in PC [28]. Likewise, we found a positive correlation between TIAM2 and NSUN2, and TIAM2 silence could partially reverse the oncogenic effects of NSUN2 overexpression. Moreover, TIAM2 may mediate NSUN2-induced metastasis via regulating EMT-related proteins expression. These findings revealed a novel regulatory mechanism that NSUN2 exerts its oncogenic effect through, at least in part, upregulating TIAM2 expression in PC.

Previous studies demonstrated that m5C modification plays an important role in regulating mRNA metabolic process, including pre-mRNA splicing, mRNA export, stability and translation [29]. Mechanistic studies have determined that m5C methylation written by NSUN2 can be recognized by “m5C reader” YBX1 or ALYREF, thereby impeding degradation or enhancing nuclear export of mRNA, respectively [13, 14, 18, 21]. Chen et al. verified that YBX1 can bind the m5C methylation site in the HDGF 3’ untranslated region catalyzed by NSUN2 and stabilize *HDGF* mRNA to drive bladder cancer progression [21]. It was also reported that YBX1 could enhance mRNA stabilization by recognizing m5C-modified sites through π-π interactions to facilitate the maternal-to-zygotic transition of zebrafish [13]. Herein, we found that NSUN2 and YBX1 coregulate the stability of TIAM2 mRNA. After NUN2 silencing, the ability of YBX1 to regulate TIAM2 expression was greatly weakened, suggesting that YBX1 might be implicated in NSUN2-mediated overexpression of TIAM2. However, a clearer detailed mechanism needs to be further studied. Besides, very recently, Su and coworkers identified LIN28B as a novel m5C reader to inhibit m5C-modified *GRB2* mRNA decay in ESCC [19]. Therefore, whether other readers or m5C-related enzymes contribute to the TIAM2 expression requires further investigation.

In summary, our study revealed that NSUN2 is elevated in PC and predicts an unfavorable prognosis. We reported for the first time that NSUN2 facilitates PC progression by upregulating TIAM2 expression in an m5C-dependent manner. NSUN2 may represent a promising therapeutic target for PC treatment.

## Materials and methods

### Bioinformatics analysis

Data retrieved from The Cancer Genome Atlas (TCGA), Genotype-Tissue Expression (GTEx) and Gene Expression Omnibus (GEO) dataset was used for analysis. Gene set enrichment analysis (GSEA)was conducted with normalized data using the R language tools. Two gene sets were utilized for GSEA (HALLMARK_EPITHELIAL_MESENCHYMAL_TRANSITION,GOTZMANN_EPITHELIAL_TO_MESENCHYMAL_TRANSITION_UP).

### Human tissue specimens and cell lines

A tissue microarray (TMA) containing 90 paired pancreatic cancer and adjacent normal tissues was provided by Shanghai Outdo Biotech Co., Ltd. and another TMA containing 32 pancreatic cancer tissues was purchased from Expect laboratory (Tsingtao, China). Human pancreatic ductal epithelial cell line HPDE6C7 and PC cell lines MIA PaCa-2 and CFPAC-1 were preserved by our laboratory. SW1990, PANC-1 were kindly provided by Dr. Qiankun Luo at Zhengzhou University. HPDE6C7, SW1990, PANC-1and CFPAC-1 were cultivated in the DMEM (VivaCell, Shanghai, China) with 10% fetal bovine serum (FBS, VivaCell, Shanghai, China), whereas MIA PaCa-2 cells were maintained in DMEM with 10% FBS and 2.5% super horse serum (Sangon Biotech, Shanghai, China). All cells were cultured in a humidified 5% CO_2_ atmosphere at 37 °C.

### Lentiviral transduction and cell transfection

Lentivirus constructs were purchased from HanBio (Shanghai, China). 2 × 10^5^ cells were seeded into a six-well plate and then transfected with NSUN2 overexpression (namely NSUN2) or knockdown (shNSUN2, shYBX1) recombinant lentivirus after 24 h. Subsequently, PC cells were maintained in medium containing 3ug/ml puromycin after 72 h to select stably transfected cells for further studies. For TIAM2 silence experiments, the lentivirus-infected PC cells were transfected with small-interfering RNAs (siRNAs, RiboBio, Guangzhou, China) targeting TIAM2 (si-TIAM2) or negative control RNAs (si-Ctrl) following the manufacturer’s instructions. Nucleotide sequences for the siRNAs and shRNAs were listed in Supplementary Table 3. PCMV-YBX1WT plasmids were kind gifts from Prof. Yun-Gui Yang (Beijing Institute of Genomics, Beijing, China). Transient transfection was performed using EZ Trans cell transfection reagent (Life-iLab, Shanghai, China) according to the manufacturer’s instructions. After 48 h of culture, cells were harvested for western blotting.

### Western blot

Cells were lysed in RIPA buffer containing PMSF (Solarbio, Beijing, China). After quality control, protein samples were separated by 8% or 10% Bis-Tris SDS-PAGE gel and then transferred to the polyvinylidene fluoride membranes. Membranes were incubated with primary antibodies at 4 °C overnight, followed by incubation with the corresponding horseradish peroxidase-conjugated or fluorescent-labeled secondary antibody. Signals were detected by the enhanced chemiluminescence (ECL) (SH-Focus 523, Hangzhou, China) or fluorescence imaging system (Odyssey, Nebraska, USA). anti-GAPDH or anti-β-actin antibody was used as the internal control. The information of antibodies and all original western blot images were presented in Supplementary Table 4.

### Quantitative real-time PCR (qRT-PCR)

Total RNA isolated by TRIzol (Ambion, Texas, USA) was utilized for synthesizing complementary DNA (cDNA) by using a RevertAid First Strand cDNA Synthesis Kit (Thermo Fisher, Massachusetts, Scientific, USA). Subsequently, the complementary DNA was amplified to test TIAM2 by real-time PCR using a SYBR Green Master Mix (Servicebio, Wuhan, China). GAPDH was used as the internal control. The relative mRNA expression was quantified using 2^-ΔΔCt^ method. The PCR primers were listed in Supplementary Table 5.

### Cell viability assay

Cell viability was assessed by CCK-8 assay. Briefly, cells were seeded on 96-well plates ((3–5) × 10^3^ cells/well). The culture medium and CCK-8 solution (Dojindo, Kyushu, Japan) were mixed with a 9:1 ratio and then incubate cells for 1.5 h. The optical density (OD) values were measured at 450 nm using a Microplate reader (BioTek, Vermont, USA) at 24th, 48th, 72nd, 96th and 120th h.

### Colony formation assay

For this assay, 1000 cells/well were cultured in 6-well plates (or 500 cells/well in 12-well plates) for 12–14 days. The colonies were stained with 0.1% crystal violet (Solarbio, Beijing, China) for 10 min after fixation using paraformaldehyde for 15 min. Finally, the colonies were counted.

### 5-Ethynyl-2e′-deoxyuridine (EdU) assay

EdU staining assay was performed by EdU assay kit (RiboBio, Guanghzou, China). Briefly, cells were incubated with 25 μM EdU for 5 h and then fixed with 4% paraformaldehyde. Subsequently, cells were dyed by Apollo stain mixture and Hoechst 33342 for 30 min, respectively. A fluorescence microscope (Olympus, Tokyo, Japan) was utilized to take images to calculate the proportion of EdU-positive cells.

### Transwell migration and invasion assays

Transwell chambers (Corning, New York, USA) were used for these assays. For invasion assays, the Matrigel (Corning, New York, USA) was thawed at 4 °C overnight. 100 µL Matrigel diluted by serum-free medium was added to the upper chamber and incubated for 30 min for solidification at 37 °C. Next, 3 × 10^4^ cells suspended in 200 µL serum-free medium were placed to the upper chamber, and 600 µL 10% FBS-supplemented medium to the lower chamber. After 36-h incubation at 37 °C, both chambers were immersed in 10% formaldehyde and dyed with 0.1% crystal violet for 20 min, respectively, and then cells in the upper chamber were wiped off with cotton swabs. Finally, stained cells on the lower membrane surface were counted under a microscope. Transwell migration experiment shared the same protocols with invasion assays but without 100 µL Matrigel in the upper chambers.

### m5C quantification

The global m5C level was assessed using the MethylFlash™ 5-mC RNA Methylation ELISA Easy Kit (Fluorometric) (EpiGentek, New York, USA). Briefly, adding 100 µl binding solution and 200 ng sample total RNA into each well, followed by incubation at 37 °C for 90 min for RNA binding. Then, 50 µl of m5C Detection Complex Solution containing m5C antibody was applied into each well after washing. Subsequently, diluted m5C antibody were removed after 50-min incubation at room temperature. Finally, wells were incubated with Fluorescence Development Solution at room temperature for 2–4 min away from direct light. The signal was determined by a fluorescence microplate reader within 2 to 10 min at 530ex/590em nm.

### RNA-seq and m5C-methylated RNA immunoprecipitation sequencing(m5C-seq)

Cloudseq Biotech Inc. (Shanghai, China) provided RNA-seq and m5C-seq service. RNA-seq was conducted as previously described [45]. For m5C-seq, NanoDrop ND-1000 (Thermo Fisher Scientific, Massachusetts, USA) was firstly used to evaluate the quality and quantity of total RNA, followed by integrity evaluation using denaturing agarose. If OD260/OD280 values range from 1.8 to 2.1, the RNA purity is qualified and the RNA extracted from all samples met this standard. Then, RNA was randomly fragmented into fragments of about 200 nt and subsequently incubated with m5C antibody (NEB, Massachusetts, USA) for immunoprecipitation. Immunoprecipitated RNA was analyzed by high-throughput sequencing on the NovaSeq 6000 sequencer (Illumina, California, USA) to generate raw reads. Then, paired-end reads harvested from Novaseq 6000 sequencer were quality controlled by Q30 and a Q30 > 80% indicates good sequencing quality. Following removal of 3′ adaptor-trimming and inferior quality reads, clean reads were aligned to the reference genome (UCSC HG38) by Hisat2 software (v2.0.4) [46]. Methylated sites on RNAs were identified using MACS software [47]. Differentially methylated sites (|fold change | ≥2 and *p* < 0.000001) were selected by diffReps [48]. The pathway enrichment analysis was conducted on the differentially methylated sites-harboring genes based on Kyoto Encyclopedia of Genes and Genomes (KEGG) database. *P* < 0.05 was statistically significant.

### mRNA stability assays

5 μg/ml actinomycin D (ActD, GlpBio, California, USA) were added to treat PC cells for 0, 4,8 h. Then, samples were harvested for total RNA extraction. qRT-PCR was performed to analyze the remaining *TIAM2* mRNA expression. GAPDH was used as the internal control.

### Animal experiment

The 4–6-week-old male BALB/c nude mice provided by Ziyuan Eperimental Animal Technology Co., Ltd. (Hangzhou China) were housed in specific pathogen-free units. Mice were humanely sacrificed by cervical dislocation after anesthesia at the endpoint of the experiment. Animal studies were approved by the ethics committee of the First Affiliated Hospital of Zheng Zhou University.

For the subcutaneous model, mice were randomly assigned into each group according to the random table method (*n* = 5 per group). 5 × 10^6^ SW1990(transfected with shCtrl or shNSUN2) or PANC-1 cells (transfected with Vector or NSUN2) were subcutaneously injected into the lower flanks of mice to establish the xenograft tumors. Tumor weight and volume were recorded subsequently and tumor tissues were collected for Hematoxylin and eosin (HE)-staining and immunohistochemistry (IHC). Tumor volume (mm^3^) = (width)^2^ × length/2.

To evaluate the peritoneal dissemination ability, 1 × 10^6^ transfected PC cells were injected into the peritoneal cavity of mice (*n* = 5/6 per group). Mice were carefully monitored and sacrificed at the 30th day after injection. Tissue with metastatic nodules was examined histopathologically.

For construction of hematogenous metastasis model, 1 × 10^6^ transfected PC cells were injected into the tail veins of mice (*n* = 5/6 per group). Mice were sacrificed at the endpoint. Lungs and livers were collected for histopathological examination.

### Immunohistochemistry (IHC) and evaluation

IHC and evaluation were performed as previously described [49]. The score of 11 and 12 was defined as high expression while the others as low expression for statistical analysis. Detailed information of antibodies for IHC was shown in Supplementary Table 4.

### Statistical analysis

Statistical analysis was performed by SPSS Statistics 23.0 (IBM, New York, USA) and GraphPad Prism software8.0.1 (GraphPad, California, USA). Differences between two groups were evaluated by a two-tailed Student’s t test, χ2 test or Fisher’s exact test. Spearman’s correlation analysis was utilized to estimate the correlation between two genes. Survival analysis was conducted by Kaplan–Meier and log-rank test. Cox’s proportional hazard regression model was used for identifying significant independent prognostic factors. *P* < 0.05 (two-tailed) was statistically significant.

## Supplementary information

Supplementary FigureS5

Supplementary FigureS6

Supplementary FigureS7

Supplementary FigureS8

Supplementary figure legends

Supplementary FigureS1

Supplementary FigureS2

Supplementary FigureS3

Supplementary FigureS4

Supplementary Table1

Supplementary Table2

Supplementary Table3

Supplementary Table4

Supplementary Table5

Supplementary Table6

Original Data File

PANC-1-STR

MIA PaCa-2-STR

SW 1990-STR

CFPAC-1-STR

## Data Availability

The authors declared that all the data and materials are available on reasonable request.
