# Supplementary figures and images for: NSUN2 stimulates tumor progression via enhancing TIAM2 mRNA stability in pancreatic cancer
Source: Cell Death Discov. 2023 Jul 1;9:219. doi: 10.1038/s41420-023-01521-y (PMC10314926; doi:10.1038/s41420-023-01521-y)

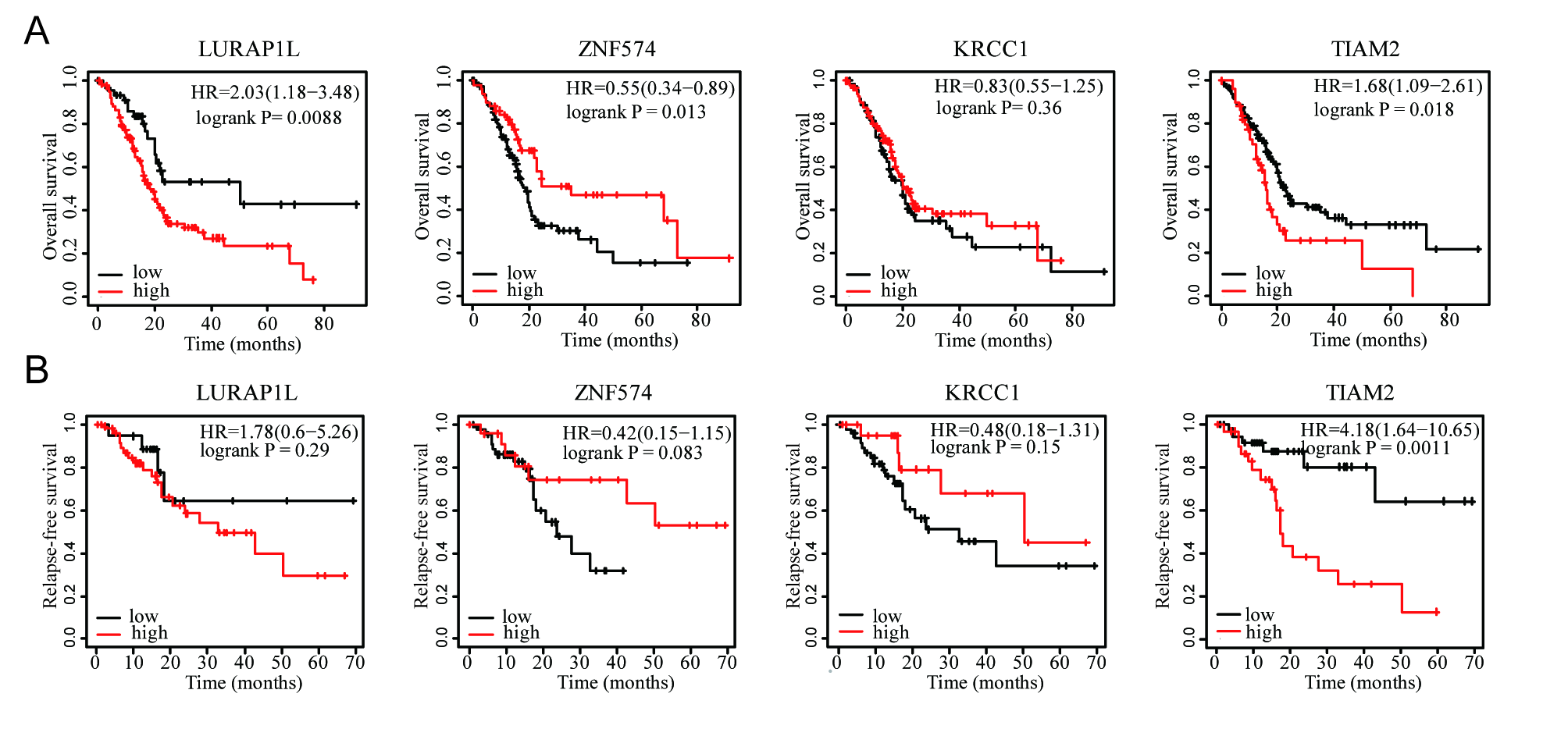

Supplement: Supplementary file 1 — Supplementary FigureS5 [file 41420_2023_1521_MOESM1_ESM.tif]

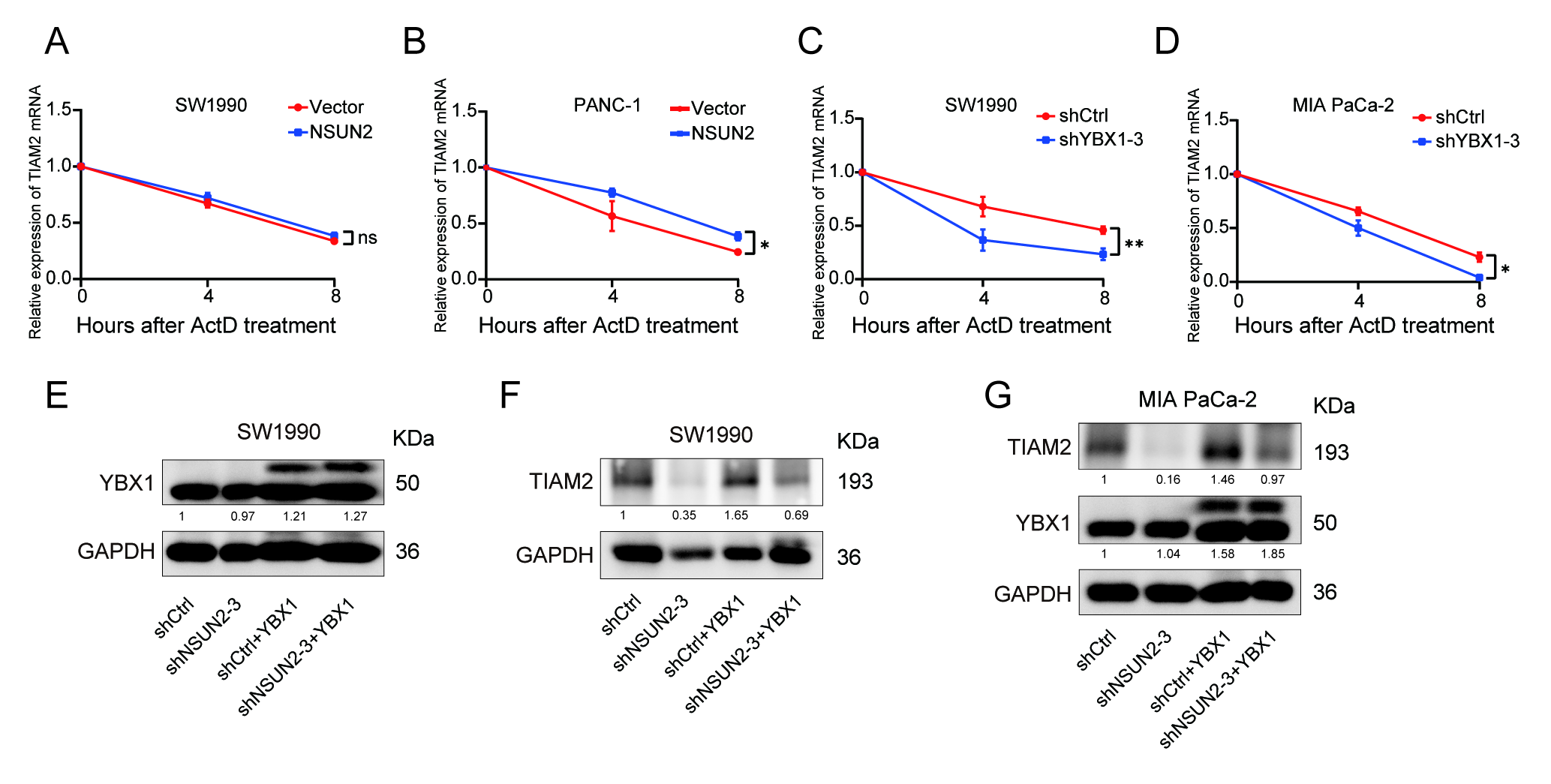

Supplement: Supplementary file 2 — Supplementary FigureS6 [file 41420_2023_1521_MOESM2_ESM.tif]

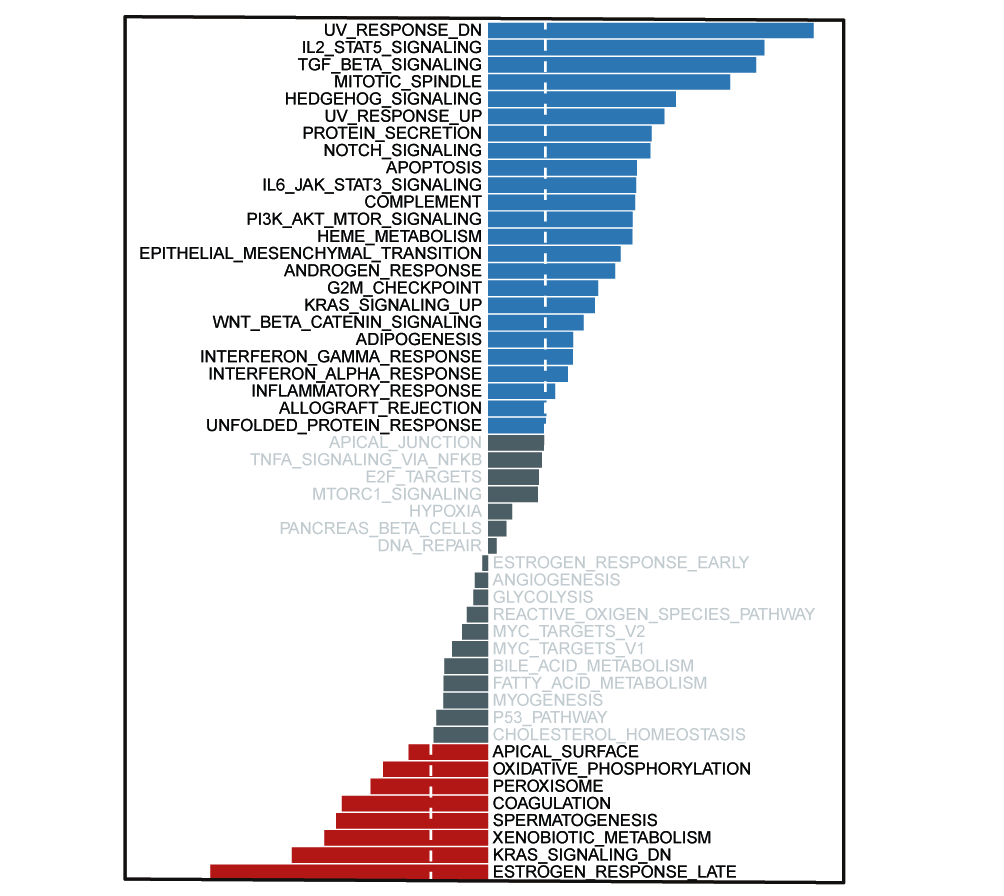

Supplement: Supplementary file 3 — Supplementary FigureS7 [file 41420_2023_1521_MOESM3_ESM.tif]

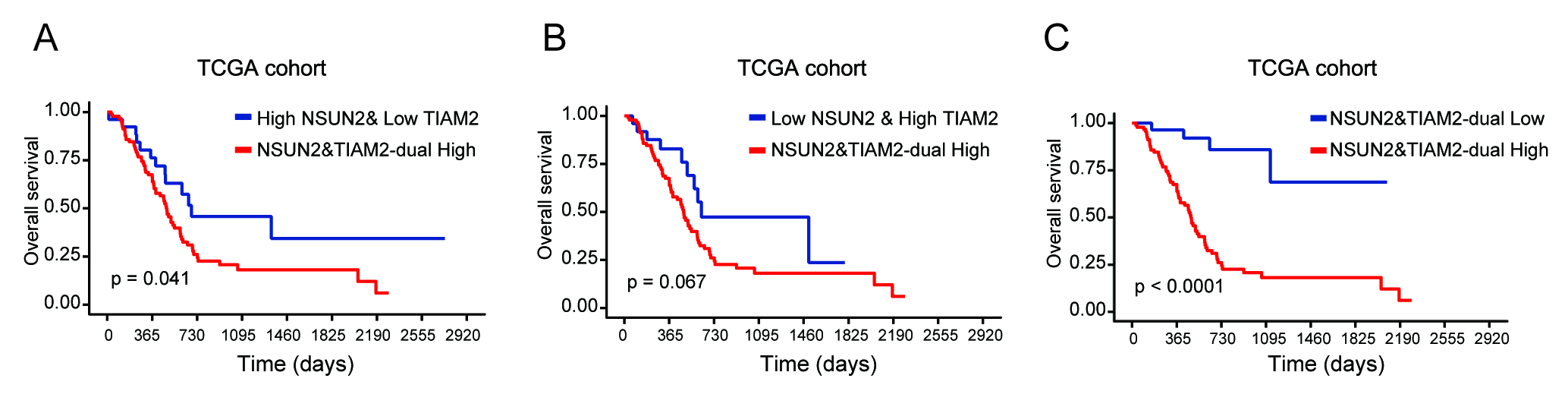

Supplement: Supplementary file 4 — Supplementary FigureS8 [file 41420_2023_1521_MOESM4_ESM.tif]

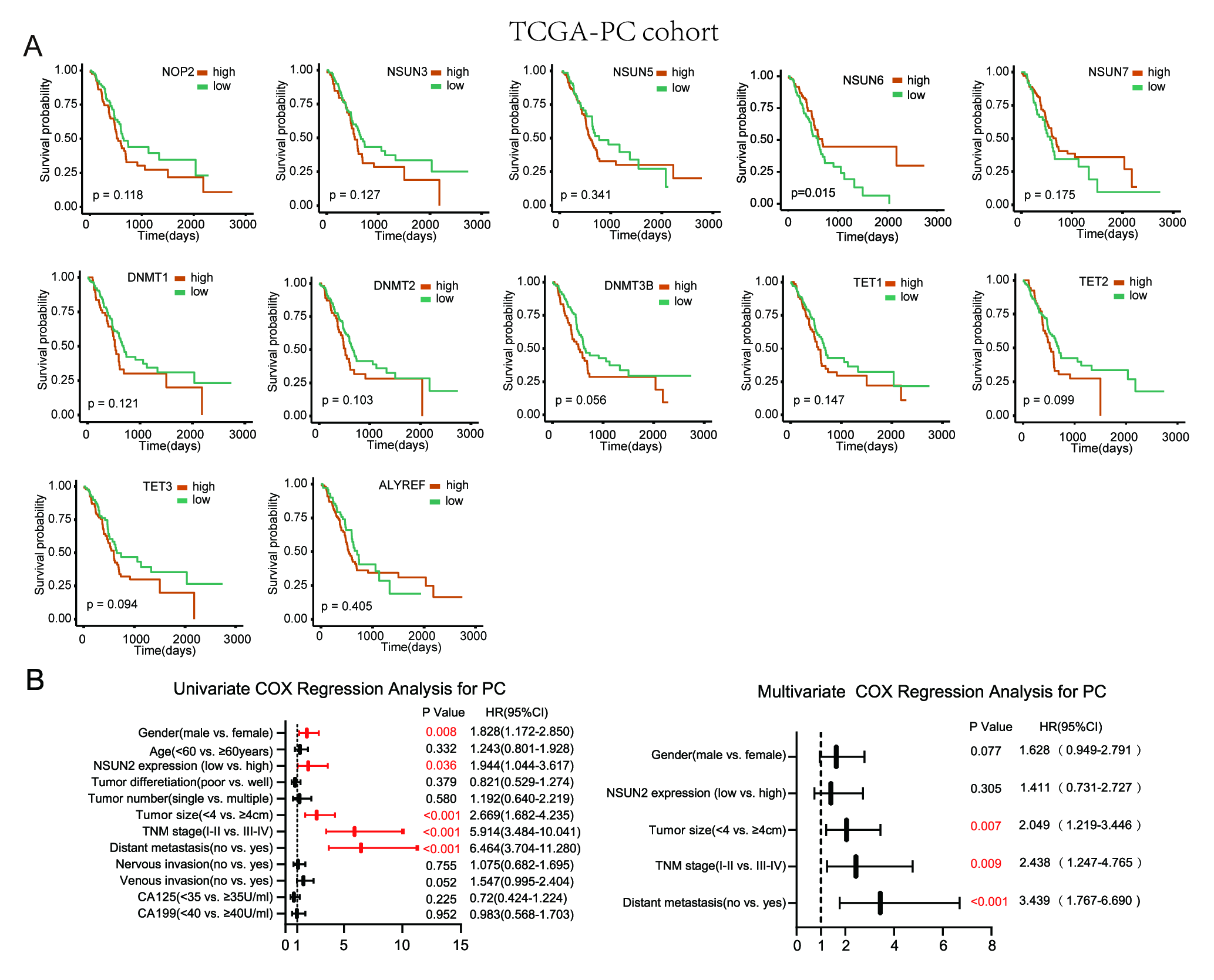

Supplement: Supplementary file 6 — Supplementary FigureS1 [file 41420_2023_1521_MOESM6_ESM.tif]

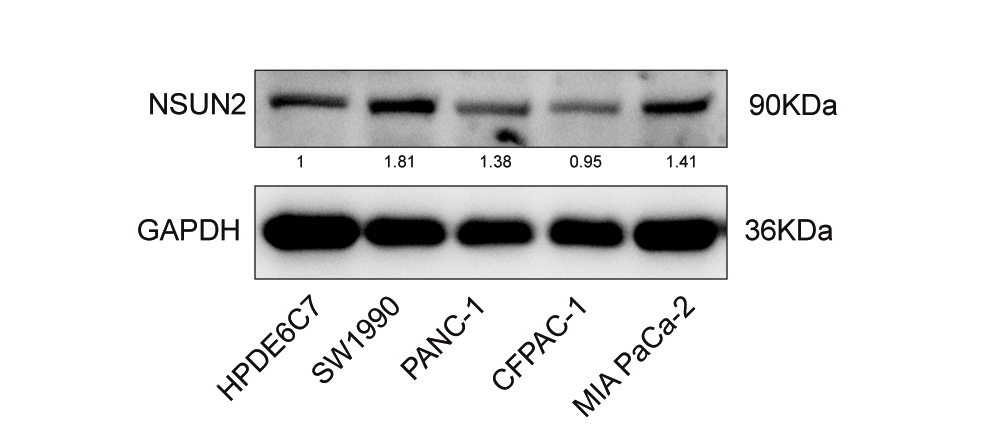

Supplement: Supplementary file 7 — Supplementary FigureS2 [file 41420_2023_1521_MOESM7_ESM.tif]

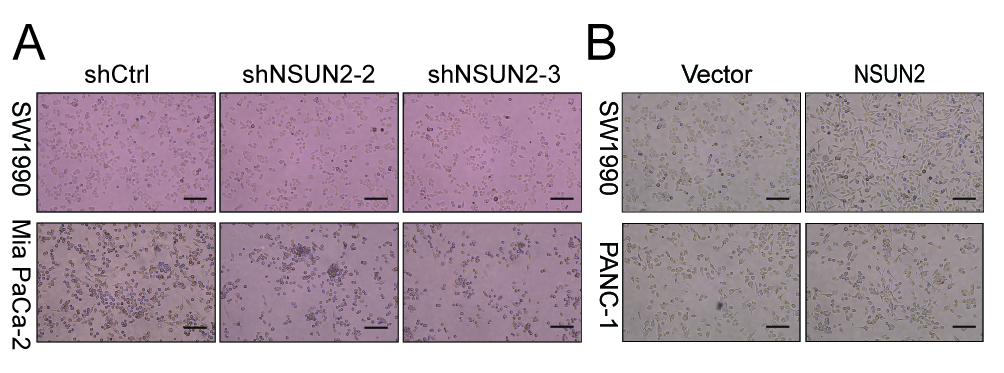

Supplement: Supplementary file 8 — Supplementary FigureS3 [file 41420_2023_1521_MOESM8_ESM.tif]

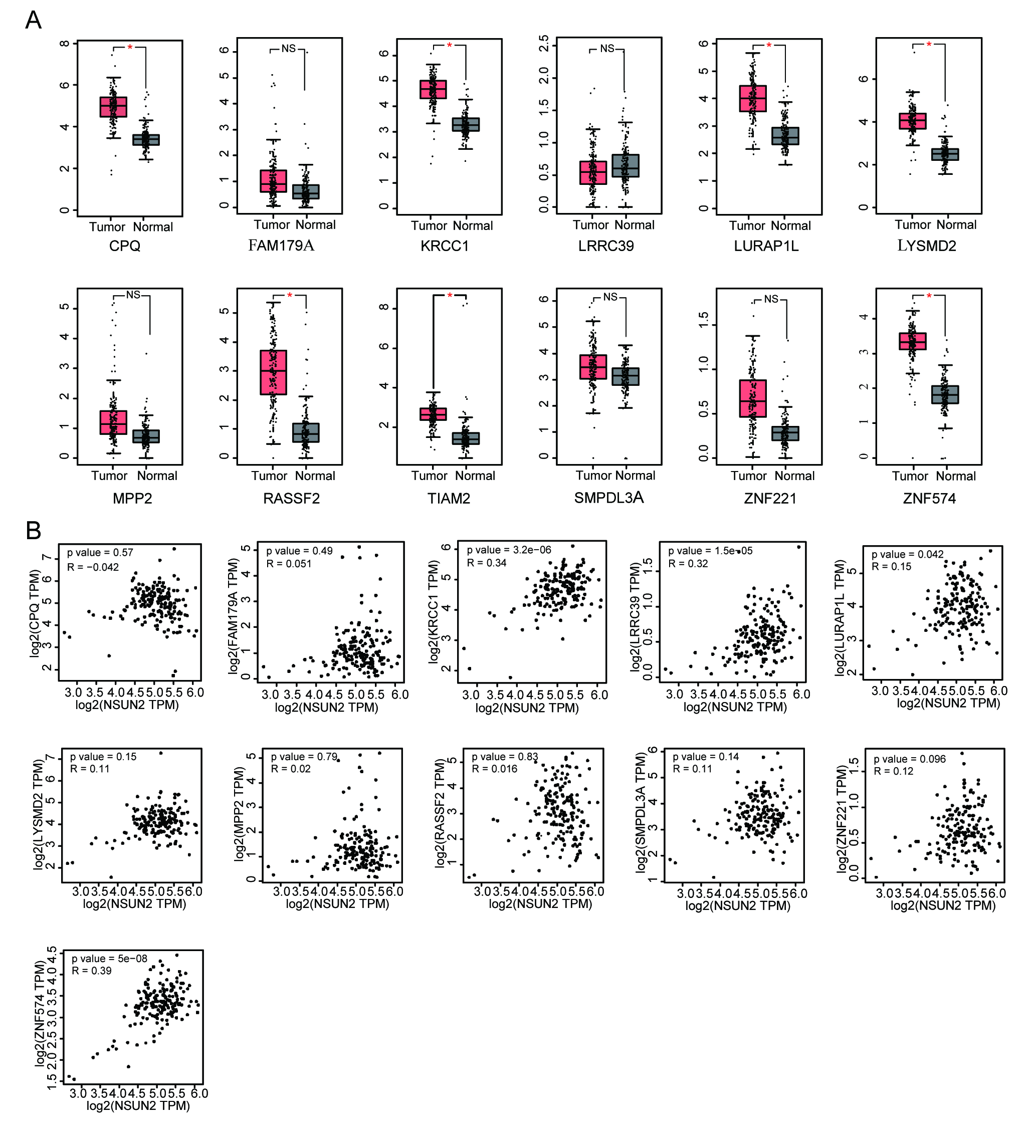

Supplement: Supplementary file 9 — Supplementary FigureS4 [file 41420_2023_1521_MOESM9_ESM.tif]

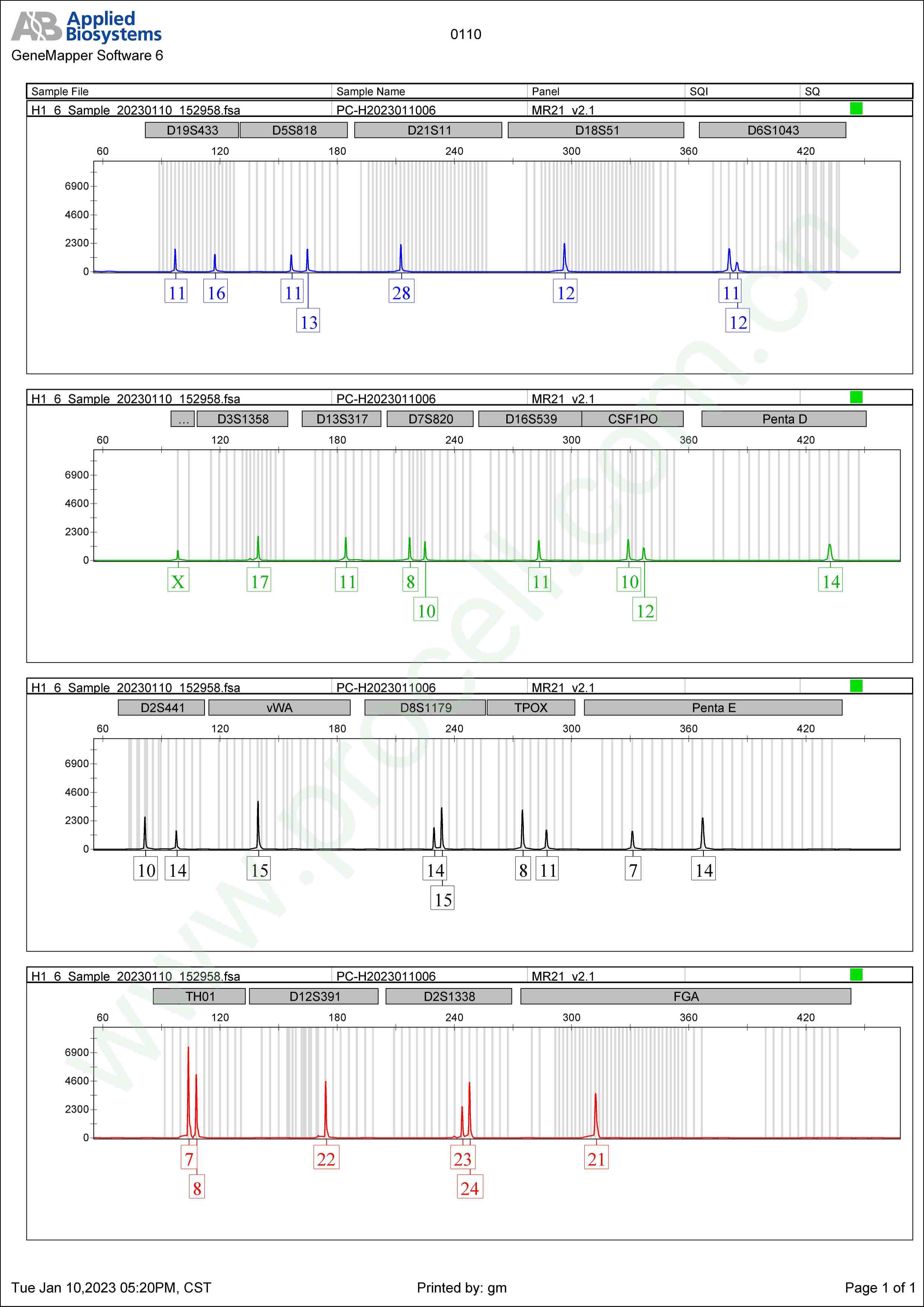

Supplement: Supplementary file 17 — PANC-1-STR [file 41420_2023_1521_MOESM17_ESM.jpg]

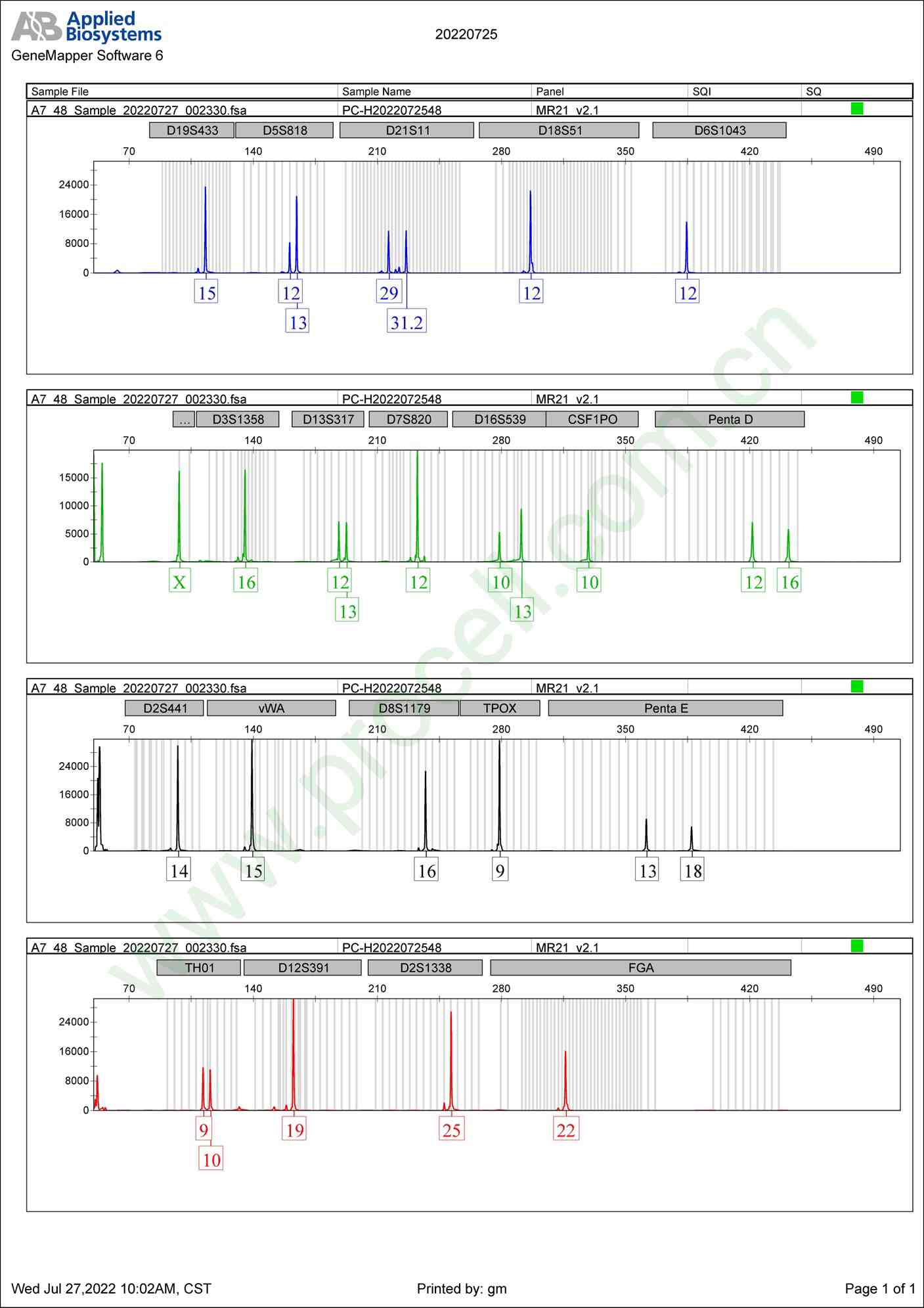

Supplement: Supplementary file 18 — MIA PaCa-2-STR [file 41420_2023_1521_MOESM18_ESM.jpg]

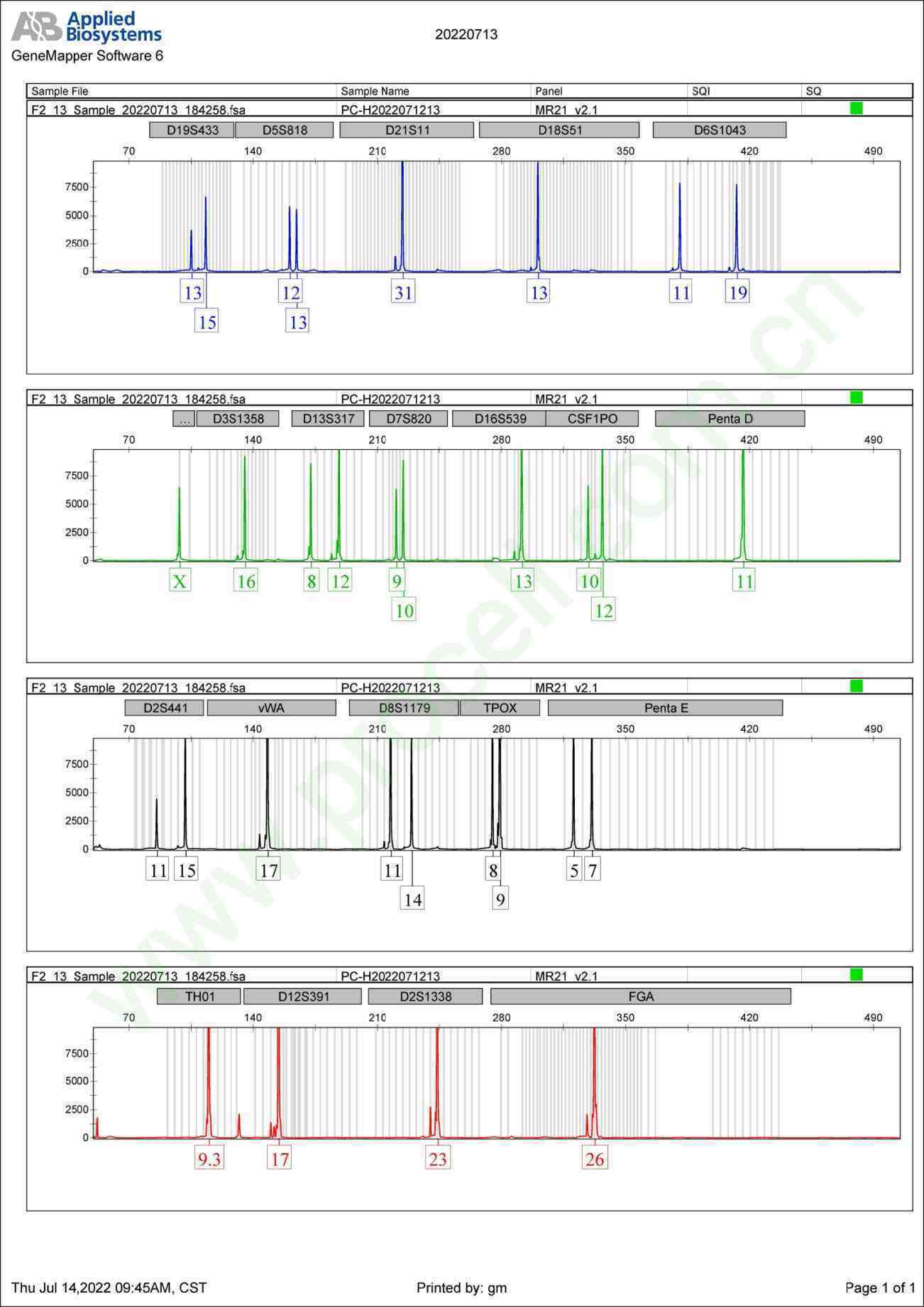

Supplement: Supplementary file 19 — SW 1990-STR [file 41420_2023_1521_MOESM19_ESM.jpg]

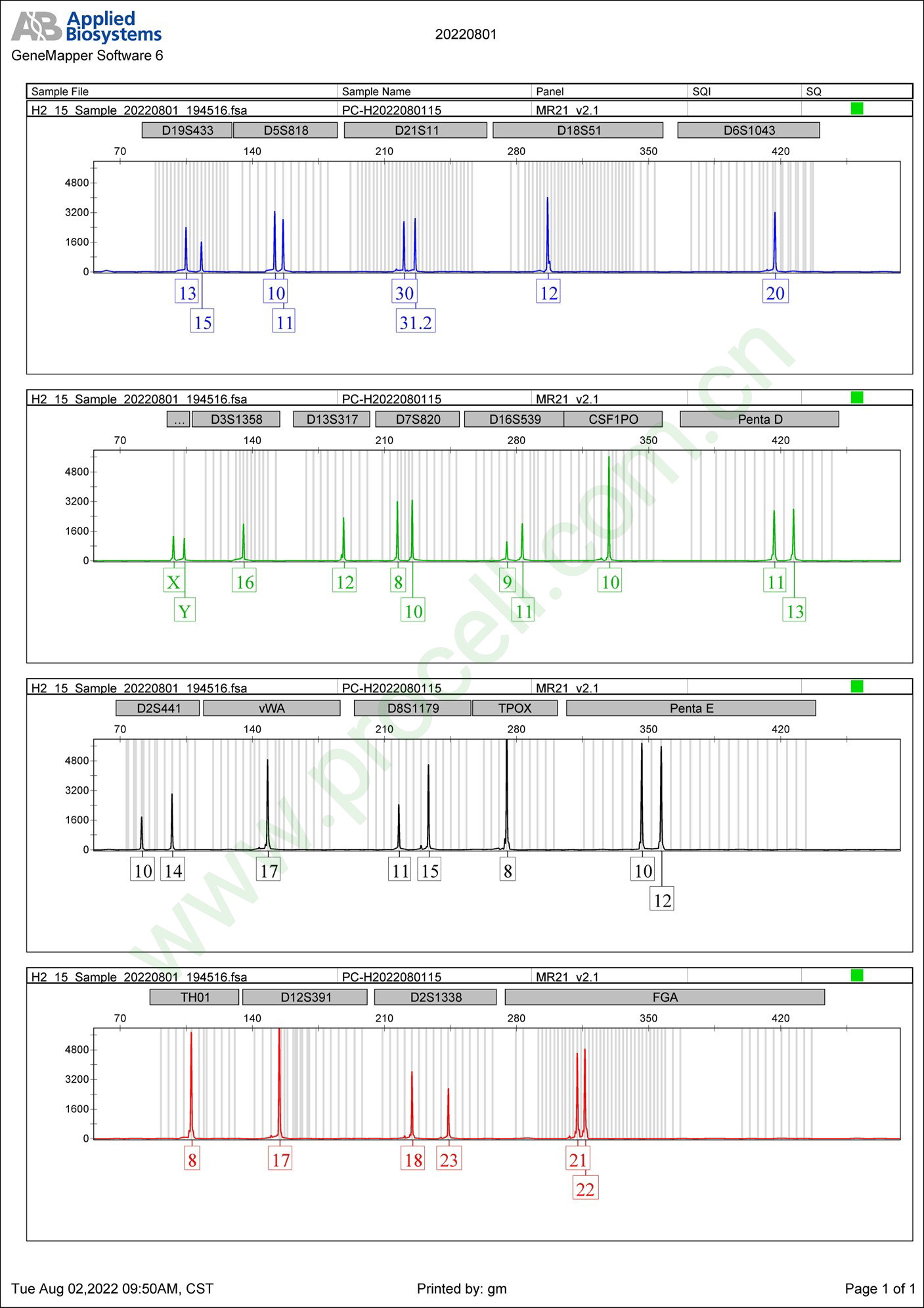

Supplement: Supplementary file 20 — CFPAC-1-STR [file 41420_2023_1521_MOESM20_ESM.jpg]
