# Supplementary material for: NSUN2 stimulates tumor progression via enhancing TIAM2 mRNA stability in pancreatic cancer
Source: Cell Death Discov. 2023 Jul 1;9:219. doi: 10.1038/s41420-023-01521-y (PMC10314926; doi:10.1038/s41420-023-01521-y)
Supplement: Supplementary file 5 — Supplementary figure legends [file 41420_2023_1521_MOESM5_ESM.docx]

**Supplementary figure legends**

**Fig.S1 Clinical significance of m5C-related regulators in human pancreatic cancer. A** The prognostic value of m5C-related genes for OS of PC patients in TCGA cohort. **B** Univariate and multivariate COX regression analyses for prognosis of pancreatic cancer. TNM, tumor-node- metastasis. HR, hazard ratio.

**Fig.S2 NSUN2 expression in HPDE6C7 and pancreatic cancer cell lines**. Western blot images were analyzed by ImageJ software.

**Fig.S3 NSUN2 promoted morphological mesenchymal transformations in pancreatic cancer cells. A** PC cells exhibited a cobblestone shape when NSUN2was silenced. **B** PC cells with overexpressed NSUN2 showed a spindle-like morphology. Scale bar=100μm.

**Fig.S4 TIAM2 was identified as a downstream target of NSUN2. A** The mRNA expression of twelve potential targets of NSUN2 in PC tissues compared with non-tumor tissues based on TCGA and GTEx database. **B** The correlations of twelve potential target genes expression with NSUN2 expression were studied base on TCGA data. NS, not significant.

**Fig.S5 Prognostic value of candidate genes in PC patients. A, B** The graph shows the results of Kaplan-Meier analysis of the overall survival (OS) rate (**A**) and relapse-freee survival (RFS) rate (**B**) in PC patients in the TCGA database with high or low expression of candidate genes. Survival analysis was conducted on the platform of Kaplan–Meier Plotter (kmplot.com/analysis).

**Fig.S6 TIAM2 expression was coregulated by NSUN2 and YBX1. A, B** TIAM2 mRNA stability in SW1990 (**A**) and PANC-1 (**B**) cells with overexpressed NSUN2. **C, D** TIAM2 mRNA stability in SW1990 (**C**) and PANC-1 (**D**) cells transduced with shYBX1. Cells were treated with 5 μg/ml actinomycin D for indicated times. Data are presented as the mean ± SD of 2 or 3 replicates. *P < 0.05, **P < 0.01. ns，not significant. **E-G** Western blot analysis of TIAM2 expression after NSUN2 silence and/or YBX1 overexpression in SW1990(**E, F**) and MIA PaCa-2 cells(**G**). Western blot images were analyzed by ImageJ software.

**Fig.S7 KEGG analysis was performed to identify the enriched pathways in TIAM2-high or -low patients with pancreatic cancer groups in TCGA.**

**Fig.S8 Survival analysis of pancreatic cancer patients with different NSUN2 and TIAM2 expression status.**
