# Supplementary material for: NSUN2 stimulates tumor progression via enhancing TIAM2 mRNA stability in pancreatic cancer
Source: Cell Death Discov. 2023 Jul 1;9:219. doi: 10.1038/s41420-023-01521-y (PMC10314926; doi:10.1038/s41420-023-01521-y)
Supplement: Supplementary file 10 — Supplementary Table1 [file 41420_2023_1521_MOESM10_ESM.docx]

| **variable** |  | **Univariate** | | ***P* value** | **Multivariate** | | ***P* value** |
| --- | --- | --- | --- | --- | --- | --- | --- |
|  | **n** | **HR** | **95% CI** |  | **HR** | **95% CI** |  |
| **Gender** |  |  |  | **0.008*** |  |  | 0.077 |
| Male | 53 |  |  |  |  |  |  |
| Female | 37 | 1.828 | （1.172-2.85） |  | 1.628 | (0.949-2.791) |  |
| **Age(years)** |  |  |  | 0.332 |  |  |  |
| <60 | 47 |  |  |  |  |  |  |
| ≥60 | 43 | 1.243 | （0.801-1.928） |  |  |  |  |
| **Tumor differentiation** |  |  |  | 0.379 |  |  |  |
| Poor | 37 |  |  |  |  |  |  |
| Well | 53 | 0.821 | （0.529-1.274） |  |  |  |  |
| **Tumor size(cm)** |  |  |  | **<0.001*** |  |  | **0.007*** |
| <4 | 43 |  |  |  |  |  |  |
| ≥4 | 46 | 2.669 | (1.682-4.235) |  | 2.049 | (1.219-3.446) |  |
| **Tumor number** |  |  |  | 0.58 |  |  |  |
| Single | 50 |  |  |  |  |  |  |
| Multiple | 14 | 1.192 | （0.64-2.219） |  |  |  |  |
| **TNM stage** |  |  |  | **<0.001*** |  |  | **0.009*** |
| I-II | 53 |  |  |  |  |  |  |
| III～IV | 37 | 5.914 | (3.484-10.041) |  | 2.438 | (1.247-4.765) |  |
| **Distant metastasis** |  |  |  | **<0.001*** |  |  | **<0.001*** |
| No | 66 |  |  |  |  |  |  |
| Yes | 24 | 6.464 | （3.704-11.28） |  | 3.439 | (1.767-6.690) |  |
| **Nervous invasion** |  |  |  | 0.755 |  |  |  |
| Negative | 31 |  |  |  |  |  |  |
| Positive | 58 | 1.075 | （0.682-1.695） |  |  |  |  |
| **Venous invasion** |  |  |  | 0.052 |  |  |  |
| No | 52 |  |  |  |  |  |  |
| Yes | 37 | 1.547 | （0.995-2.404） |  |  |  |  |
| **NSUN2 expression** |  |  |  | **0.036*** |  |  | 0.305 |
| Low | 15 |  |  |  |  |  |  |
| High | 67 | 1.944 | (1.044-3.617) |  | 1.411 | (0.731-2.727) |  |
| **CA199**（**U/ml**） |  |  |  | 0.952 |  |  |  |
| <40 | 17 |  |  |  |  |  |  |
| ≥40 | 69 | 0.983 | (0.568-1.703） |  |  |  |  |
| **CA125**（**U/ml**） |  |  |  | 0.225 |  |  |  |
| <35 | 47 |  |  |  |  |  |  |
| ≥35 | 22 | 0.72 | （0.424-1.224） |  |  |  |  |
