# Supplementary material for: NSUN2 stimulates tumor progression via enhancing TIAM2 mRNA stability in pancreatic cancer
Source: Cell Death Discov. 2023 Jul 1;9:219. doi: 10.1038/s41420-023-01521-y (PMC10314926; doi:10.1038/s41420-023-01521-y)

Figure 3

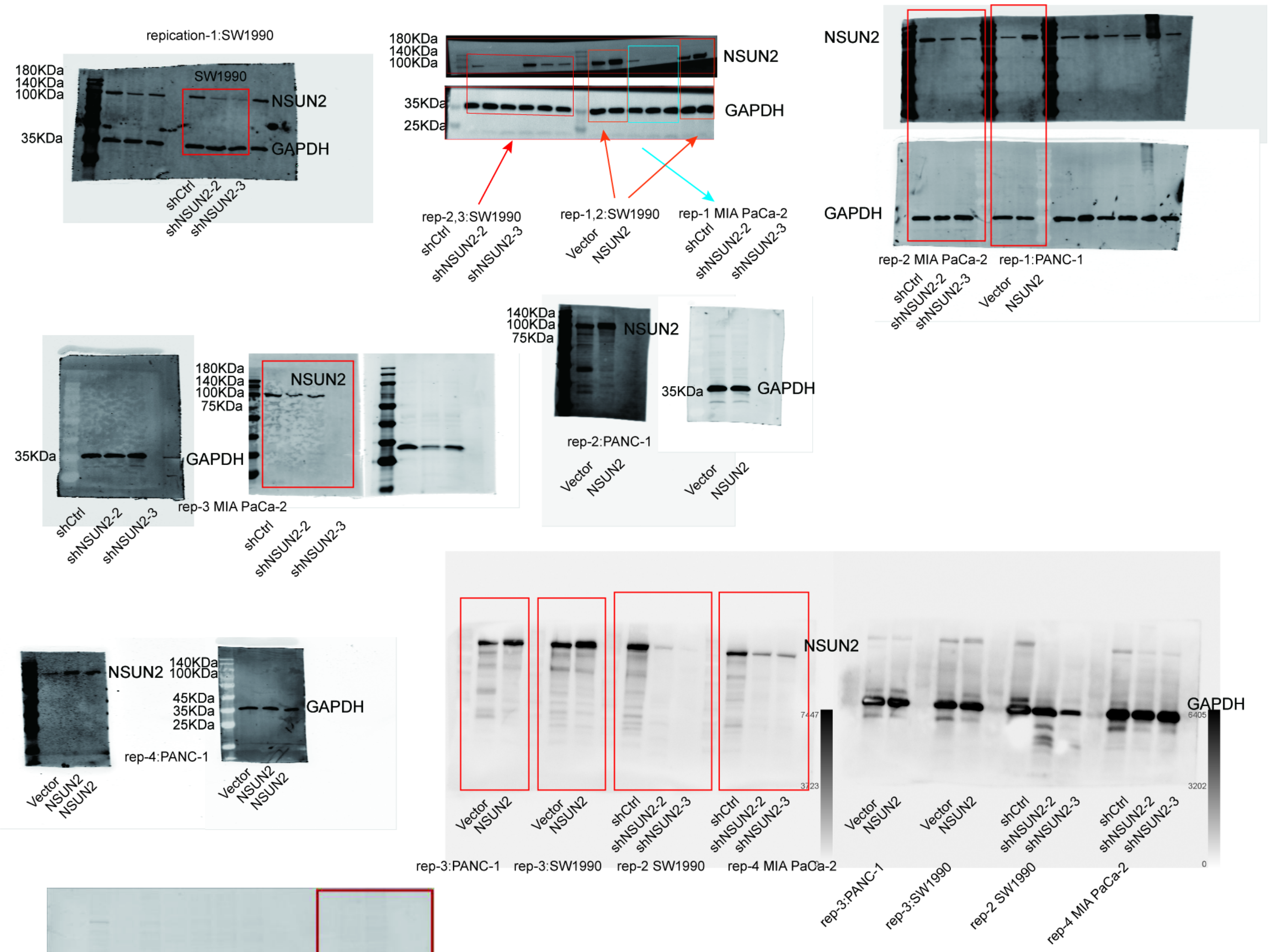

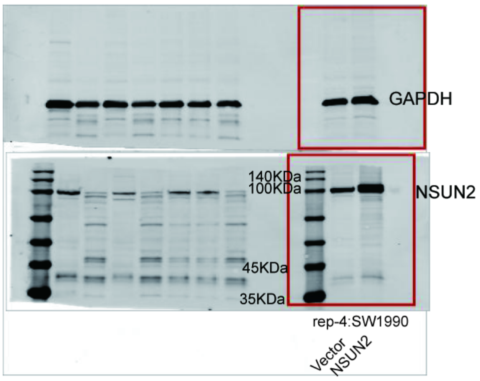

Figure S2

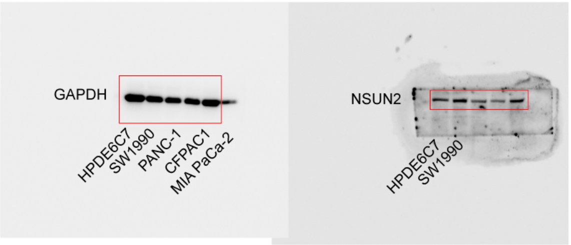

Figure 4 Figure 6

SW1990 cells

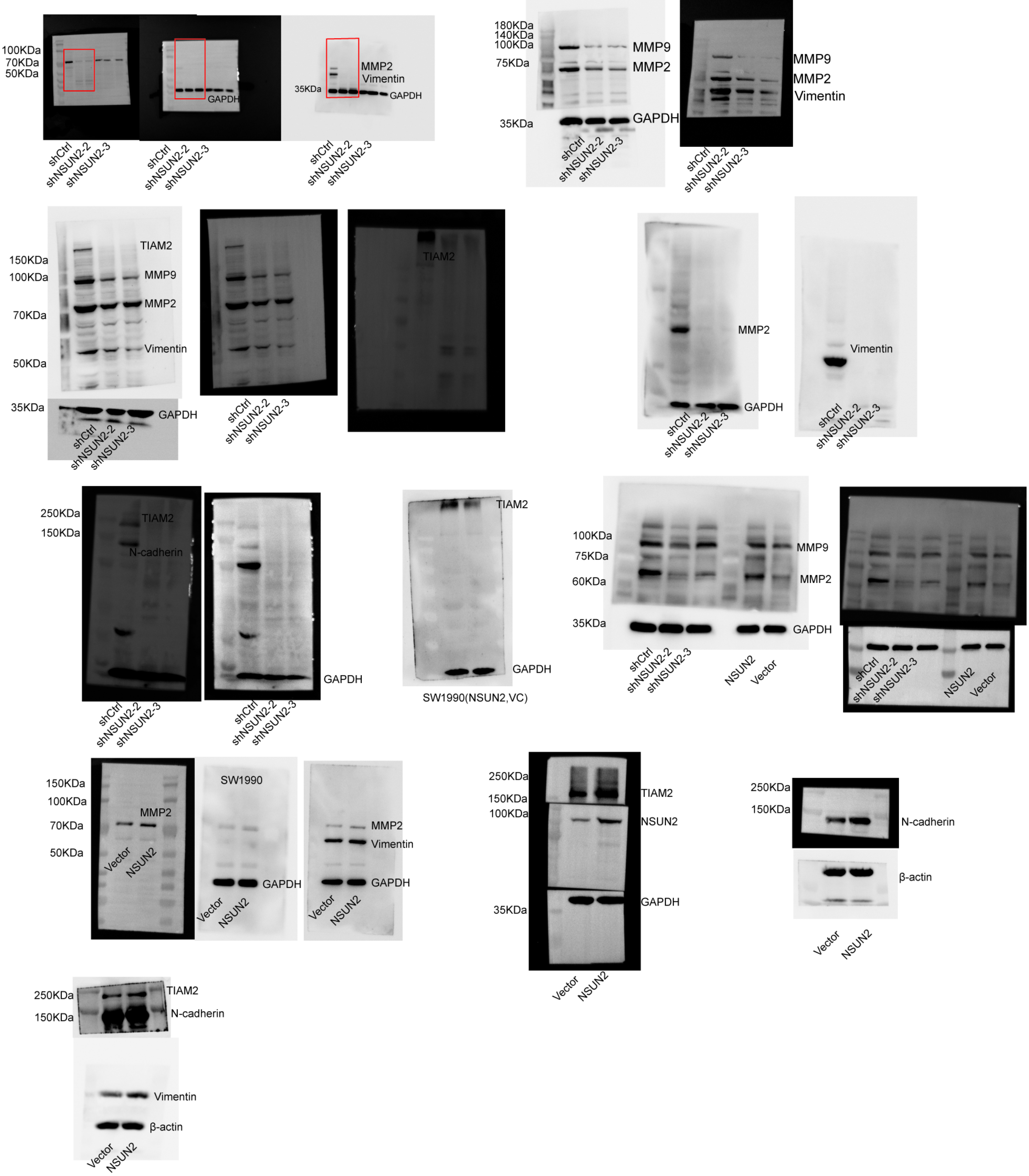

Figure 4 Figure 6

PANC-1

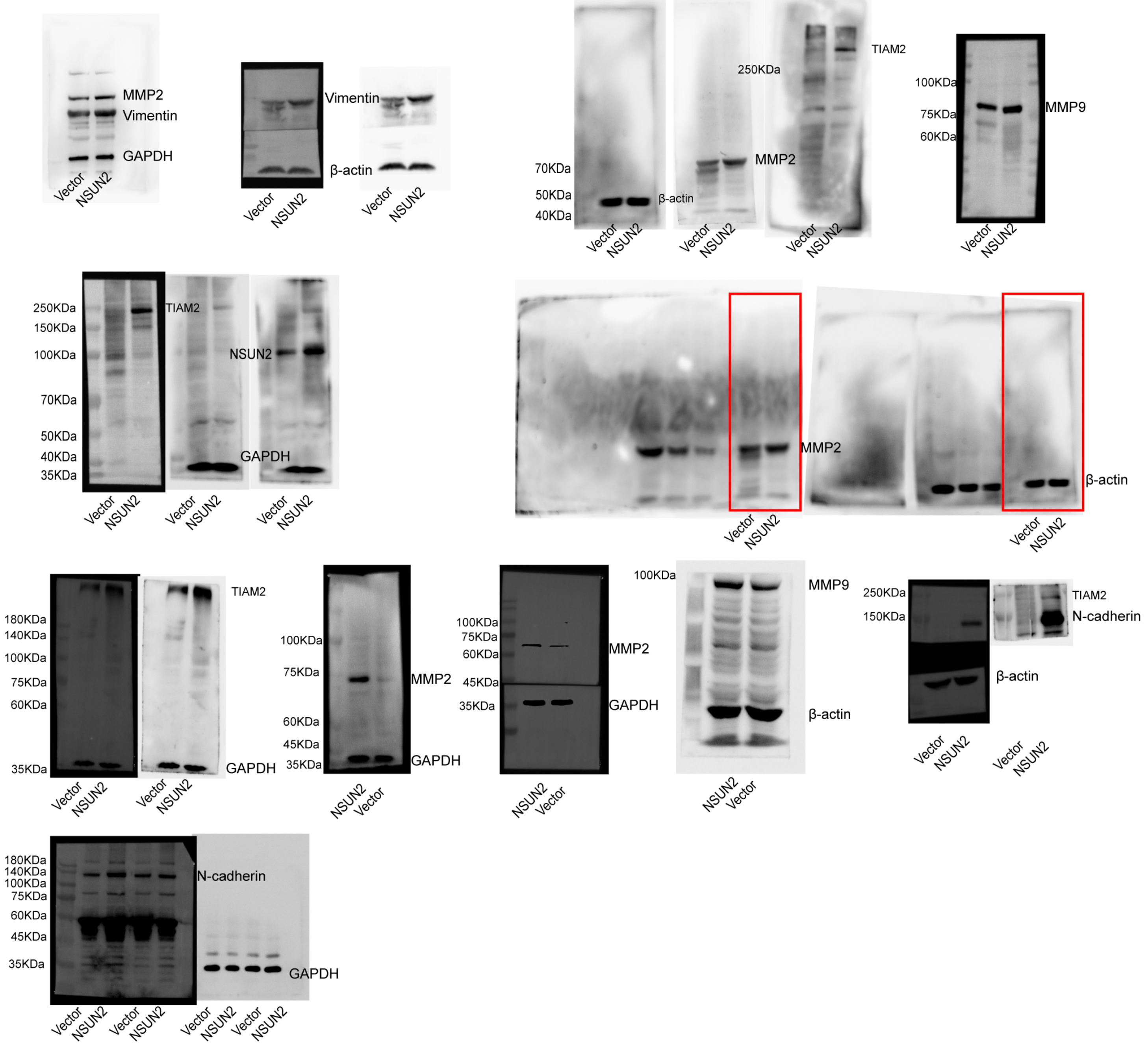

Figure 4 Figure 6

MIAPaCa-2

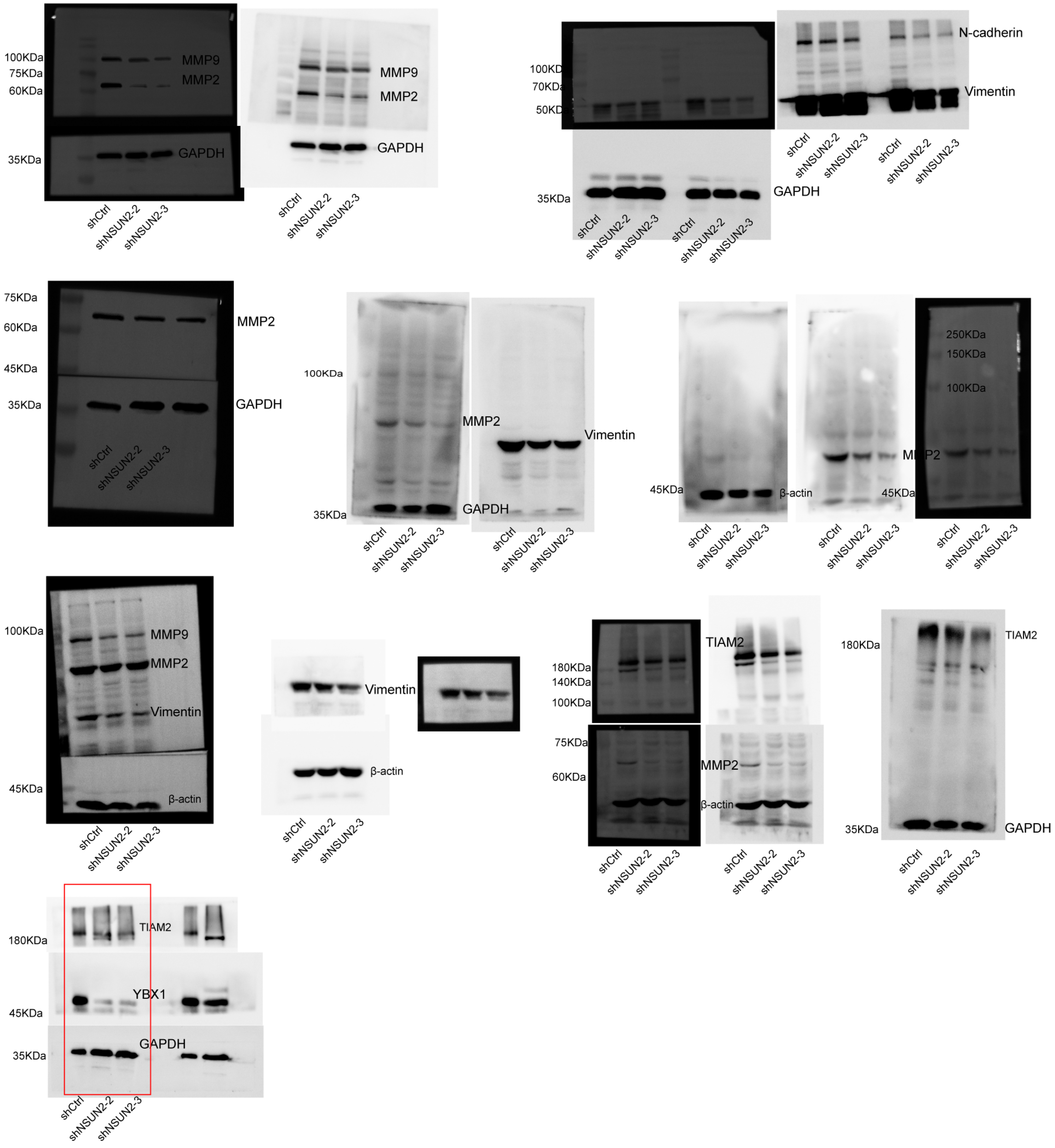

Figure7

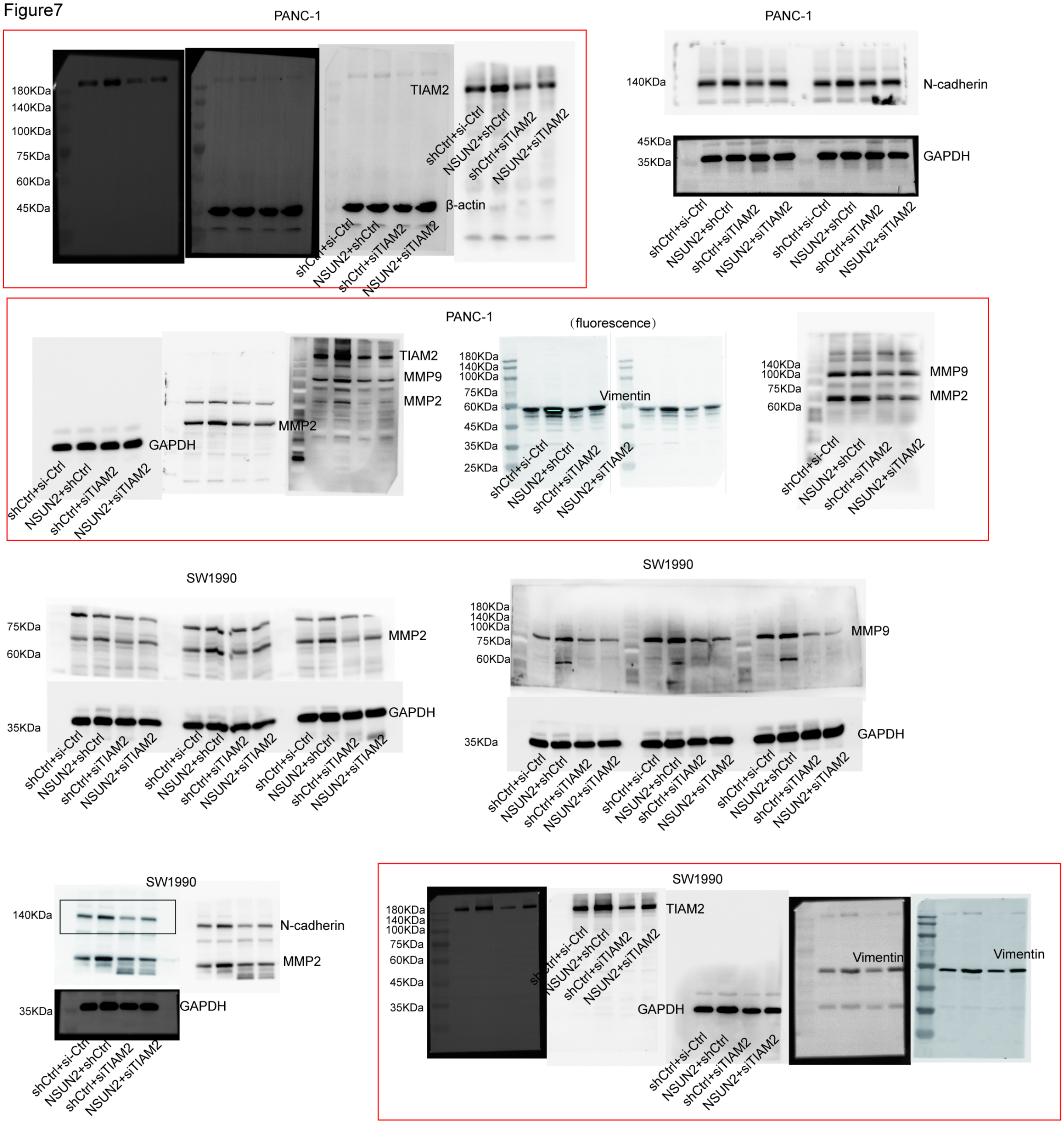
PANC-1
180KDa
140KDa
100KDa
75KDa
60KDa
45KDa
TIAM2
β-actin
shCtrl+si-Ctrl
NSUN2+shCtrl
shCtrl+siTIAM2
NSUN2+siTIAM2
PANC-1
140KDa
N-cadherin
45KDa
35KDa
GAPDH
PANC-1
（fluorescence）
TIAM2
MMP9
MMP2
MMP2
GAPDH
Vimentin
25KDa
MMP9
MMP2
SW1990
MMP2
GAPDH
SW1990
MMP9
GAPDH
SW1990
N-cadherin
MMP2
GAPDH
SW1990
TIAM2
Vimentin
Vimentin
GAPDH

FigureS6

SW1990

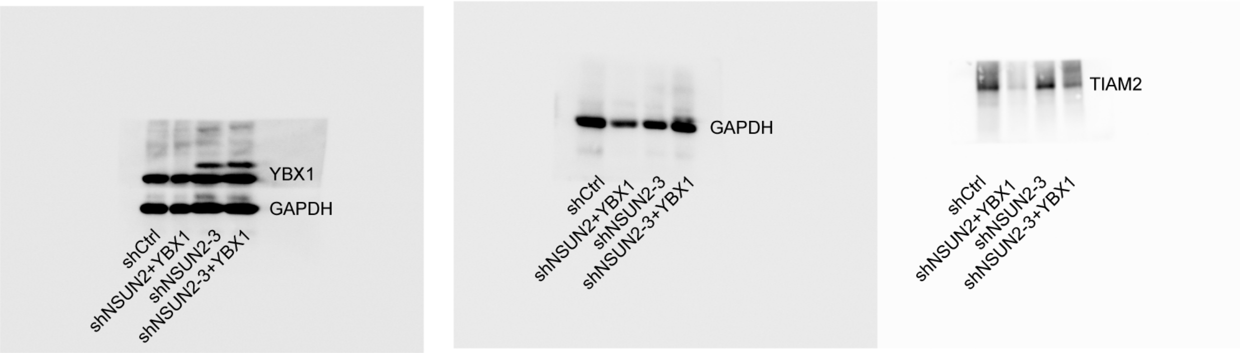

MIA PaCa-2

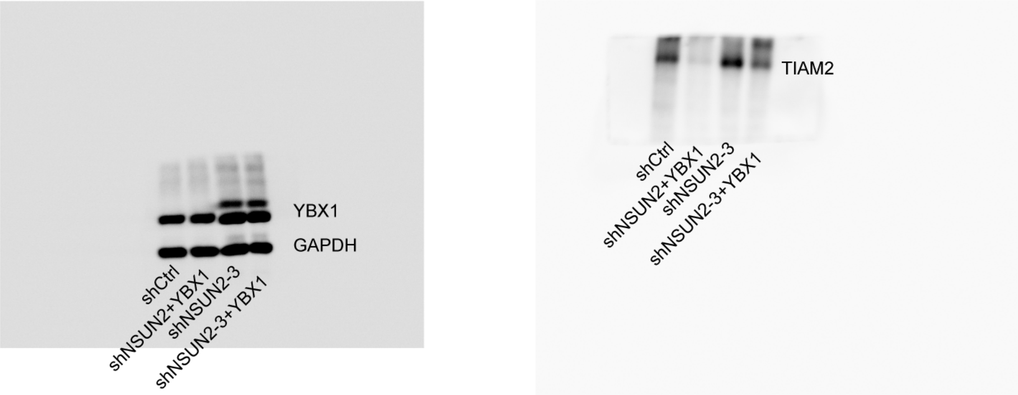

Supplement: Supplementary file 16 — Original Data File [file 41420_2023_1521_MOESM16_ESM.pdf]
